# Limited Impact of the Protein Corona on the Cellular Uptake of PEGylated Zein Micelles by Melanoma Cancer Cells

**DOI:** 10.3390/pharmaceutics14020439

**Published:** 2022-02-18

**Authors:** Jitkasem Meewan, Sukrut Somani, Partha Laskar, Craig Irving, Margaret Mullin, Stuart Woods, Craig W. Roberts, Abdullah R. Alzahrani, Valerie A. Ferro, Suzanne McGill, Stefan Weidt, Richard Burchmore, Christine Dufès

**Affiliations:** 1 Strathclyde Institute of Pharmacy and Biomedical Sciences, University of Strathclyde, 161 Cathedral Street, Glasgow G4 0RE, UK; jitkasem.meewan@strath.ac.uk (J.M.); sukrut.somani@biontech.de (S.S.); partha.laskar@utrgv.edu (P.L.); stuart.woods@strath.ac.uk (S.W.); c.w.roberts@strath.ac.uk (C.W.R.); aralzahrani@uqu.edu.sa (A.R.A.); v.a.ferro@strath.ac.uk (V.A.F.); 2 Department of Immunology and Microbiology, University of Texas Health Rio Grande Valley, 5300 North L Street 881 Madison, McAllen, TX 78504, USA; 3 Department of Pure and Applied Chemistry, University of Strathclyde, 295 Cathedral Street, Glasgow G1 1XL, UK; craig.irving@strath.ac.uk; 4 Glasgow Imaging Facility, College of Medical, Veterinary and Life Sciences, University of Glasgow, Glasgow G12 8QQ, UK; margaret.mullin@glasgow.ac.uk; 5 Department of Pharmacology & Toxicology, Faculty of Medicine, Umm Al-Qura University, Al-Abidiyah, P.O. Box 13578, Makkah 21955, Saudi Arabia; 6 Glasgow Polyomics, Wolfson Wohl Cancer Research Centre, Garscube Campus, College of Medical, Veterinary and Life Sciences, University of Glasgow, Glasgow G61 1QH, UK; suzanne.eadie@glasgow.ac.uk (S.M.); stefan.weidt@glasgow.ac.uk (S.W.); richard.burchmore@glasgow.ac.uk (R.B.)

**Keywords:** zein micelles, poly(ethylene glycol), protein corona, cellular uptake, cancer cells, macrophages, dendritic cells

## Abstract

The formation of a protein layer “corona” on the nanoparticle surface upon entry into a biological environment was shown to strongly influence the interactions with cells, especially affecting the uptake of nanomedicines. In this work, we present the impact of the protein corona on the uptake of PEGylated zein micelles by cancer cells, macrophages, and dendritic cells. Zein was successfully conjugated with poly(ethylene glycol) (PEG) of varying chain lengths (5K and 10K) and assembled into micelles. Our results demonstrate that PEGylation conferred stealth effects to the zein micelles. The presence of human plasma did not impact the uptake levels of the micelles by melanoma cancer cells, regardless of the PEG chain length used. In contrast, it decreased the uptake by macrophages and dendritic cells. These results therefore make PEGylated zein micelles promising as potential drug delivery systems for cancer therapy.

## 1. Introduction

Zein, a prolamin protein extracted from corn, has been widely used in food, pharmaceutical, and biomedical applications because of its generally regarded as safe (GRAS) status [1,2,3]. It is classified into four main types, depending on their molecular weight (MW), charge, and solubility [4]. Its major fraction, α-zein, consists of 75–80% of the total zein with a MW of 19–24 kDa, while β-zein is a polymer of 17–18 kDa. γ-zein contains two parts of 27 kDa and 18 kDa, and δ-zein is a minor fraction of 10 kDa [5,6,7,8]. Zein exhibits an amphiphilic molecular structure. It contains more than 50% lipophilic amino acids, leading to insolubility in water. Its high glutamine content also makes it insoluble in absolute alcohol [9], but facilitates conjugation with biomolecules via its N-terminal group. Due to its good biocompatibility and biodegradability, zein has shown high potential in carrier systems for the delivery of nutraceutical and drugs [10,11,12,13,14,15] and for use in cancer research [16,17].

However, because of their hydrophobicity and protein nature, zein particles may cause immunogenicity, which could limit their use for drug delivery [18]. To overcome this issue, the modification of zein by conjugation with poly(ethylene glycol) (PEG) has been proposed to create a steric shielding of the delivery system, thus reducing opsonization and providing a sustained release of the carried drug. Podaralla et al. and Song et al. previously demonstrated that PEGylation of white zein (containing a very low level of xanthophylls (less than 0.001%)) could generate stable micelles that improved the solubility and stability of the hydrophobic drug curcumin as well as enhancing its uptake by cancer cells [19,20]. So far, there have been no reports of PEGylation to yellow zein (a mixture of α, β, γ, and δ-zein, containing 8–9% of xanthophyll pigments, including lutein, zeaxanthin, and β-cryptoxanthin).

It is well known that physicochemical properties of delivery systems (e.g., size, shape, charge, and surface chemistry) strongly influence their interactions with cells [21]. However, when exposed to biological fluids, nanoparticles tend to interact with biomolecules, leading to the formation of a complex layer of proteins called a protein corona. Protein corona can be classified into “hard corona” and “soft corona” depending on binding affinity and the time required for the exchange of adsorbed proteins. The hard corona corresponds to the innermost layer of proteins that interact directly with the nanomaterial surface with high affinity and require a high exchange time (about several hours). In contrast, the soft corona is made of loosely bound proteins that adsorb with low affinity and can undergo a rapid exchange of biomolecules within seconds or minutes [22,23]. The presence of the protein corona significantly changes the surface properties of the nanocarrier, providing a new biological identity which impacts its actual biological responses within the body, such as cellular uptake, kinetics, and toxicity [23,24].

Although PEGylation is often used to prevent nonspecific protein adsorption on delivery systems [25,26], little is known about the impact of the protein corona on PEGylated zein micelles and their interactions with cells, or about the PEG chain length required to obtain stealth zein micelles. The aims of this project were therefore (1) to synthesize zein micelles conjugated with low and high MW methoxy PEG (mPEG) and entrap the hydrophobic drug model Nile red, (2) to assess the impact of PEG MW on their uptake efficiency in melanoma cancer cells, and (3) to investigate the effect of protein corona surrounding these micelles upon contact with human plasma on their cellular uptake in vitro on cancer cells, macrophages, and dendritic cells.

## 2. Materials and Methods

### 2.1. Cell Lines and Reagents

Yellow zein, Nile red, and all other chemicals that are not specifically mentioned below were purchased from Sigma-Aldrich (Poole, UK). Methoxy PEG succinimidyl carboxymethyl ester with a MW of 5 kDa (mPEG-SCM-5000) and 10 kDa (mPEG-SCM-10K) were obtained from JenKem Technology (Plano, TX, USA). Dulbecco’s Modified Eagle Medium (DMEM) and Roswell Park Memorial Institute (RPMI) 1640 cell culture media, L-glutamine, fetal bovine serum (FBS), penicillin-streptomycin, and TrypLE^®^ Express came from Life Technologies (Paisley, UK). 4–20% Mini-PROTEAN^®^ TGX^TM^ gels, Precision Plus Protein^TM^ Dual Color Standards, 10× TGS buffer, 2× Laemmli sample buffer, and Silver Stain Plus^TM^ Kit were purchased from Bio-Rad (Watford, UK). Vectashield^®^ mounting medium containing 4′,6-diamidino-2-phenylindole (DAPI) came from Vector Laboratories (Peterborough, UK). Bioware^®^ B16-F10-luc-G5 mouse melanoma cells were purchased from Caliper Life Sciences (Hopkinton, MA, USA).

### 2.2. Synthesis and Characterization of PEGylated Zein

The synthesis of PEGylated zein was adapted from a method published by Podaralla et al. [19]. Briefly, yellow zein (0.1 g) was dissolved in 4 mL of 90% (*v*/*v*) ethanol. Different amounts of mPEG-SCM (0.05 g of mPEG-SCM (5 kDa) for mPEG5K-zein; 0.1 g of mPEG-SCM (10 kDa) for mPEG10K-zein) were dissolved in 1 mL of 90% (*v*/*v*) ethanol. PEG and zein solutions were mixed under stirring (100 rpm, Fisherbrand^®^ magnetic stirrer, Thermo Fisher Scientific, Waltham, MA, USA) for 3 h at 25 °C. One mL of glycine solution (1 M) was added to stop the reaction, followed by the addition of 5 mL citrate buffer (pH 7.4) to precipitate the PEGylated zein. Free PEG and ethanol were removed by dialysis (MW cut-off: 12–14 kDa) against distilled water (2.5 L) under continuous stirring (120 rpm) at 25 °C for 48 h. The distilled water was changed 3 times during the dialysis process. The resulting product was then lyophilized using a Christ Epsilon 2–4 LSC^®^ freeze dryer (Osterode am Harz, Germany). The obtained mPEG5K-zein and mPEG10K-zein were stored at −20 °C. Their composition is summarized in Table 1.

To confirm the PEGylation of zein, samples were directly analyzed by Attenuated Total Reflection—Fourier Transform Infrared spectroscopy (ATR–FTIR). The ATR–FTIR spectrum of each sample was taken with a Bruker Tensor II-FTIR^®^ spectrophotometer (Billerica, MA, USA), using 100 scans with 2 cm^−1^ resolution. Data were collected within a range of 400–4000 cm^−1^ at 20 °C.

### 2.3. Preparation of mPEG-Zein Micelles Encapsulating Nile Red

Nile red (0.25 mg) and mPEG-zein (50 mg) were dissolved in 10 mL of ethanol (90%, *v*/*v*). To allow partitioning of the Nile red into mPEG-zein micelles, the mixture was stirred (100 rpm) at 37 °C overnight, before being dialyzed and lyophilized as described in Section 2.2.

### 2.4. Characterization of mPEG-Zein Micelles

#### 2.4.1. Characterization of Core-Shell Structure of mPEG-Zein Micelles

To characterize the core-shell structure of the micelles, mPEG-zein conjugates (5 mg/mL in deuterium oxide (D_2_O) and deuterated dimethyl sulfoxide (DMSO-d_6_)) were analyzed by Proton Nuclear Magnetic Resonance (^1^H NMR). ^1^H NMR spectra were recorded at 500 MHz using a Bruker Avance^®^ III HD500 NMR spectrometer (Billerica, MA, USA), and the core-shell structure was confirmed by comparing the spectra in DMSO-d_6_ and D_2_O.

#### 2.4.2. Determination of Critical Micelle Concentration (CMC)

The CMC of mPEG-zein micelles was determined using Nile red as a hydrophobic fluorescent probe. Briefly, 32 µL Nile red solution (1 mg/mL in methanol) was added to plastic vials. After complete evaporation of methanol, 1 mL mPEG5K-zein and mPEG10K-zein (concentration range 0.001–1 mg/mL in 5% (*w*/*v*) glucose solution) was added to the vials. Each sample was vortexed (using a Fisherbrand^®^ vortex mixer, Thermo Fisher Scientific, Waltham, MA, USA) and incubated overnight at 25 °C before measurement. Fluorescence intensity was measured with a Varian Cary Eclipse^®^ spectrofluorometer (Palo Alto, CA, USA). The excitation wavelength was fixed at 550 nm, and the emission spectra were recorded from 570 nm to 800 nm (excitation and emission slit widths: 5 and 20 nm). The ratio of Nile red emission intensity at the wavelength of maximum emission (λ_max_) in the presence of mPEG-zein (I) over the fluorescence in its absence (I_0_, in 5% (*w*/*v*) glucose solution) was plotted as a function of mPEG-zein concentration. The point at which the tangents to the two linear portions of the graph intersect was defined as the CMC.

#### 2.4.3. Morphology of mPEG-Zein Micelles

The morphology of the prepared micelles (0.4 mg/mL in distilled water) was assessed by transmission electron microscopy (TEM), using a JEOL JEM-1200EX^®^ transmission electron microscope (Jeol, Tokyo, Japan) operating at an accelerating voltage of 80 kV. Samples (3 μL) were pipetted onto a carbon-coated copper grid (400 mesh size) and were allowed to air dry overnight before imaging.

#### 2.4.4. Size and Zeta Potential Measurements

The size, polydispersity index (PDI), and zeta potential of Nile red-loaded mPEG-zein micelles were measured by dynamic light scattering (DLS), using a Malvern Zetasizer Nano-ZS^®^ at 37 °C (Malvern Instruments Ltd., Malvern, UK). All samples were freshly prepared and diluted to the concentration of 0.2 mg/mL in 5% (*w*/*v*) glucose solution (refractive index: 1.33) before measurement.

#### 2.4.5. Determination of Nile Red Encapsulation Efficiency

Nile red-loaded mPEG-zein micelles (1 mg) were dispersed in 1 mL distilled water and centrifuged at 5000 g for 14 min at 25°C using an IEC Micromax^®^ centrifuge (Thermo Scientific, Loughborough, UK). The supernatant was then discarded, and the pellet was lysed with 1 mL methanol. An aliquot was further diluted with methanol to the desired concentration range to measure the encapsulated Nile red, which was quantified by spectrofluorometry (λ_exc_: 550 nm, λ_em_: 636 nm, slit widths: 5 nm). The encapsulation efficiency (EE) was calculated as follows:

$$\mathrm{EE}\;\left(\%\right)=\frac{\mathrm{Amount}\;\mathrm{of}\;\mathrm{Nile}\;\mathrm{red}\;\mathrm{loaded}\;\mathrm{in}\;\mathrm{micelles}}{\mathrm{Amount}\;\mathrm{of}\;\mathrm{Nile}\;\mathrm{red}\;\mathrm{added}\;\mathrm{in}\;\mathrm{the}\;\mathrm{formulation}}\times 100$$

### 2.5. Cellular Uptake

#### 2.5.1. Cell Culture

B16-F10-luc-G5 cell line was cultured in RPMI 1640 medium supplemented with 10% (*v*/*v*) FBS, 1% (*v*/*v*) L-glutamine, and 0.5% (*v*/*v*) penicillin-streptomycin. Cultures were maintained at 37 °C in a 5% CO_2_ atmosphere, and the cells were sub-cultured routinely. Bone marrow-derived macrophages and dendritic cells were derived from tibia and femur of 6- to 8-week-old mice as described in the literature [27].

#### 2.5.2. Qualitative Analysis

The cellular uptake of Nile red-loaded mPEG-zein micelles was qualitatively investigated using confocal microscopy. B16-F10-luc-G5 cells were seeded on coverslips in 6-well plates (1 × 10^5^ cells/well) and allowed to adhere overnight. On the following day, the medium was removed and replaced with a fresh medium containing free Nile red or equivalent amount of Nile red loaded in mPEG-zein micelles (844 ng Nile red per well) at 37 °C for 2 h. Imaging of the cellular uptake was conducted using a Leica SP5^®^ confocal microscope (Leica, Wetzlar, Germany). Nile red was excited with the 543 nm laser line and detected at 615–660 nm. Cell nuclei were stained with Vectashield^®^ mounting medium containing DAPI, which was excited with the 405 nm laser line and detected at 415–491 nm.

#### 2.5.3. Quantitative Analysis

B16-F10-luc-G5 cells were seeded into 6-well plates at a density of 2 × 10^5^ cells/well and allowed to grow for 24 h. Uptake was studied by incubating cells with Nile red encapsulated in mPEG-zein micelles or free in solution, as described for confocal microscopy. After a 2 h incubation, adherent cells were washed and detached (using 250 µL TrypLE^®^ Express and 500 µL medium per well), followed by analysis on a FACSCanto^®^ flow cytometer using FACSDiva^®^ software (BD, Franklin Lakes, NJ, USA). At least 10,000 cells were analyzed for each sample.

#### 2.5.4. Mechanisms of Cellular Uptake

To investigate the endocytosis-mediated uptake, B16-F10-luc-G5 cells were seeded using the same protocol as in Section 2.5.3. After 24 h, the medium was removed, and the cells were pretreated with the endocytosis inhibitors chlorpromazine (20 µg/mL), filipin (5 µg/mL), and colchicine (40 µg/mL) at 37°C for 30 min. Afterward, the treatments were removed and replaced with co-incubation of Nile red-encapsulated mPEG-zein micelles (844 ng Nile red per well) with the inhibitors (chlorpromazine (5 µg/mL), filipin (3 µg/mL), and colchicine (40 µg/mL)) for another 2 h. The cells were then processed for flow cytometry analysis as described above.

### 2.6. Size and Zeta Potential of mPEG-Zein Micelles in the Presence of Human Plasma (HP)

The size and zeta potential of empty mPEG5K-zein and mPEG10K-zein micelles (1 mg/mL) in cell culture medium with 10% (*v*/*v*) HP (sfRPMI + HP) or without HP (sfRPMI) were measured at 37 °C with a Malvern Zetasizer Nano-ZS^®^ (Malvern Instruments Ltd., Malvern, UK). Samples were vortexed at the start of the experiment and were further incubated at 37 °C for subsequent measurements at the indicated times without being vortexed.

### 2.7. Effect of HP on the Cellular Uptake of mPEG-Zein Micelles

The effect of HP on mPEG-zein micelle uptake by cancer cells, macrophages, and dendritic cells was examined by flow cytometry. All three cell types were seeded into 6-well plates at a density of 2 × 10^5^ cells/well and allowed to settle for 24 h. The grown cells were then incubated in sfRPMI for 1 h, before treatment. Nile red-loaded mPEG5K-zein and mPEG10K-zein micelles (20 mg/mL in water) were pre-incubated in HP or glucose solution (5%, *w*/*v*) at a volume ratio of 1:1 at 37 °C for 1 h and were added to the cells at a concentration of 844 ng Nile red per well in serum-free medium. After 2 h treatment, cancer cells were processed for flow cytometry analysis as described in Section 2.5.3. For macrophages and dendritic cells, single cell suspensions were obtained by scraping after the addition of 500 µL medium per well.

### 2.8. Evaluation of Cell Viability

B16-F10-luc-G5 cancer cells, macrophages, and dendritic cells were seeded into 96-well plates at a density of 1.5 × 10^4^ cells/well for 24 h. Next, the cells were incubated for either 2 h (for macrophages and dendritic cells) or 4 h (for cancer cells) with Nile red loaded in mPEG-zein micelles or free as a solution (56 ng Nile red per well), using untreated cells as controls. Following treatment, 50 µL of 3-(4,5-dimethylthiazol-2-yl)-2,5-diphenyl-tetrazolium bromide (MTT) solution (5 mg/mL in PBS) was added into each well, and the cells were incubated for an additional 4 h. The MTT reagent was removed, and the formazan crystals were dissolved in 200 µL dimethyl sulfoxide. The absorbance was measured at 570 nm using a Multiskan Ascent^®^ plate reader (Thermo Scientific, Waltham, MA, USA). Cell viability was calculated as the percentage of relative change of absorbance compared with the control (untreated cells).

### 2.9. Preparation of Hard Corona Samples

HP stock solution (0.5 mL) was diluted with 2.5 mL ultrapure water. mPEG5K-zein and mPEG10K-zein micelles (3 mg) were incubated with the diluted HP (3 mL) at 37 °C for 1 h, followed by 2-step centrifugation (4696× *g*, 4 °C, 10 min) using a Heraeus Megafuge^®^ 16R centrifuge (Thermo Scientific, Loughborough, UK) to obtain hard corona pellets. The pellet was resuspended with 100 µL ultrapure water and kept at −20 °C for further study. 

### 2.10. Analysis of Protein Corona

#### 2.10.1. Sodium Dodecyl Sulfate Polyacrylamide Gel Electrophoresis (SDS-PAGE)

The protein samples were diluted with Laemmli sample buffer containing 2-mercaptoethanol (5%, *v*/*v*) as a reducing agent at the volume ratio of 1:1. The samples (5 µg protein) were reduced at 90 °C for 5 min and loaded onto a 4–20% Mini-PROTEAN^®^ TGXTM gel (Bio-Rad, Watford, UK). The gel was run with Tris/glycine/SDS buffer at 120 V for 1 h with Precision Plus Protein^TM^ Dual Color Standards (Bio-Rad, Watford, UK) as a molecular standard. The gel was stained with a Silver Stain Plus^TM^ Kit (Bio-Rad, Watford, UK), as described in the kit’s instruction manual.

#### 2.10.2. Liquid Chromatography–Mass Spectrometry (LC–MS) Analysis

The protein samples were processed by filter-aided sample preparation (FASP) to remove PEG before protein digestion by trypsin (enzyme to protein ratio of 1:100). Trypsinized peptide samples were analyzed using nanoscale liquid chromatography coupled to electrospray ionization tandem mass spectrometry (nLC-ESI–MS/MS). Online detection of peptide ion was by electrospray ionization mass spectrometry using an Orbitrap Elite^TM^ MS (Thermo Scientific, Loughborough, UK). Peptides were separated on a Pepmap^TM^ C18 reversed phase column (3 μm, 100 Å, 75 μm × 50 cm) (Thermo Scientific, Loughborough, UK). Samples were processed with mobile phase A consisting of 0.1% (*v*/*v*) formic acid in water and mobile phase B consisting of acetonitrile (80%, *v*/*v*) and water (20%, *v*/*v*). The peptide separation was performed at a fixed solvent flow rate of 0.3 µL/min, using a gradient of 4–100% mobile phase B over 120 min. The Orbitrap Elite^TM^ MS acquired full-scan spectra in the mass range of *m*/*z* 300–2000 Da for a high-resolution precursor scan at a set mass resolving power of 60,000 (at 400 *m*/*z*). Collision-induced dissociation was performed in the linear ion trap with the 20 most abundant precursors using rapid scan mode.

#### 2.10.3. Protein Identification

Data were analyzed using the Mascot search engine (v2.6.2, Matrix Science, London, UK) against the NCBIprot database using the *Homo sapiens* taxonomy. A mass tolerance of 10 ppm for the precursor and 0.3 Da MS/MS was used for peptide matching.

### 2.11. Statistical Analysis

All data were reported as means ± standard error of the mean (SEM). Statistical analysis was performed by one-way analysis of variance (ANOVA) followed by Tukey multiple comparison post-test (Minitab^®^ software, State College, PE, USA) at a significance level of 0.05.

## 3. Results and Discussion

### 3.1. Synthesis and Characterization of PEGylated Zein

**Scheme 1:**
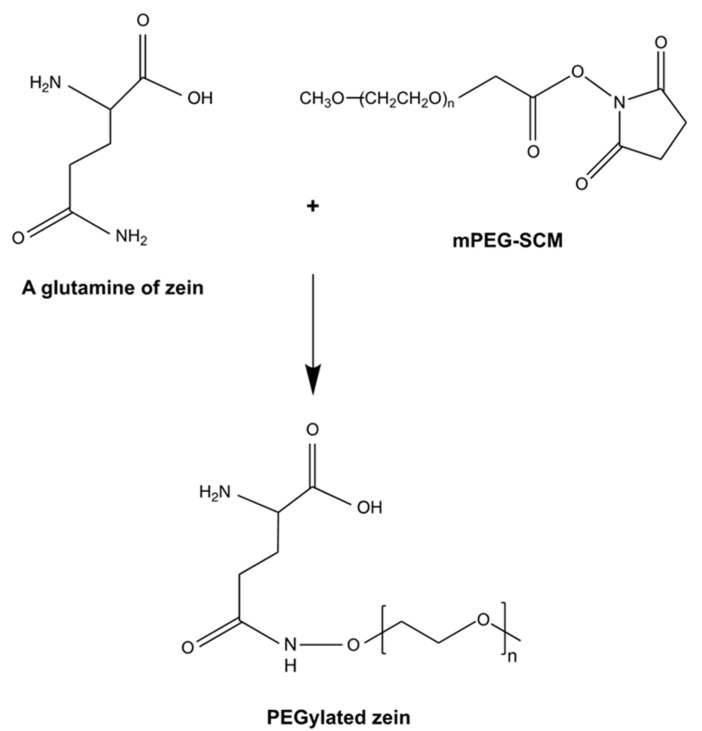
Schematic synthetic reaction for the PEGylation of zein (adapted from [19], published by American Chemical Society, 2012).

mPEG-Zein was successfully synthesized by the formation of an amide bond between the terminal amino group in yellow zein and mPEG-succinimidyl carboxymethyl (mPEG-SCM) (MW 5 and 10 kDa) (Scheme 1, Table 1). mPEG-SCM is a high-quality amine-reactive PEG product with a stable non-degradable linker between the PEG polymeric chain and the N-hydroxysuccinimide (NHS) ester. Zein contains 22% of asparagin and glutamine [9] that could theoretically be used for PEGylation due to the presence of an amino group in the side chain of these amino acids. However, these amino acids have been found to be inaccessible for conjugation [28], unlike the glutamine at the N-terminal of zein [9]. The PEGylation of zein was confirmed by ATR–FTIR analysis (Figure S1). Primary amide peaks of zein were observed at 1643 and 1516 cm^−1^ on the FTIR spectra of mPEG-zein. The stretching vibration of the carbonyl in the CH_2_CH_2_O groups of PEG at 840–960 cm^−1^ and the methyl group at 2742 cm^−1^ appeared in both the spectra of mPEG5K-zein and mPEG10K-zein. Furthermore, the NHS ester peak of mPEG at 1741 cm^−1^ disappeared after conjugation, unlike the spectra resulting from the unconjugated mixture of zein and PEG [19,20]. These demonstrated that the PEGylation of zein was successful.

### 3.2. Characterization of mPEG-Zein Micelles

**Figure 1:**
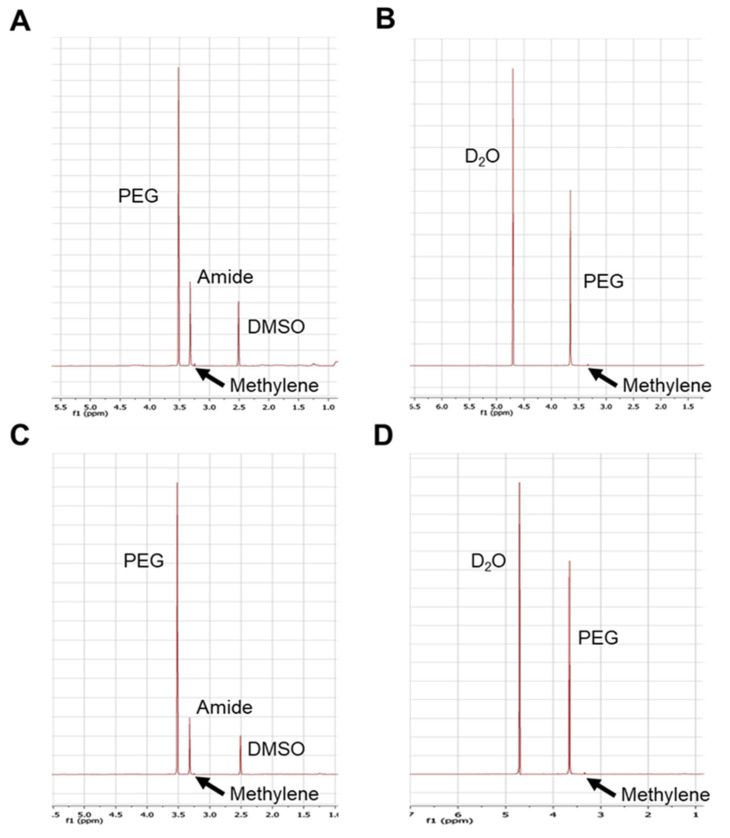
^1^H NMR spectra of mPEG5K-zein in DMSO-*d*_6_ (**A**) and in D_2_O (**B**), mPEG10K-zein in DMSO-*d*_6_ (**C**) and in D_2_O (**D**).

The assembly of mPEG-zein into micelles in water was confirmed by ^1^H NMR spectra in DMSO-d_6_ and D_2_O (Figure 1). The ethylene and methylene protons of PEG (3.5 ppm and 3.2 ppm, respectively) and the amide protons of zein (3.3 ppm) could be observed in the spectra obtained in DMSO-d_6_. However, only the PEG peaks (3.2 ppm and 3.6 ppm) were visible in the D_2_O spectra. The disappearance of the zein peak in D_2_O confirmed the amphiphilic nature of the mPEG-zein conjugate, able to assemble in water with the hydrophilic mPEG in the outer shell and the hydrophobic zein in the core.

**Figure 2:**
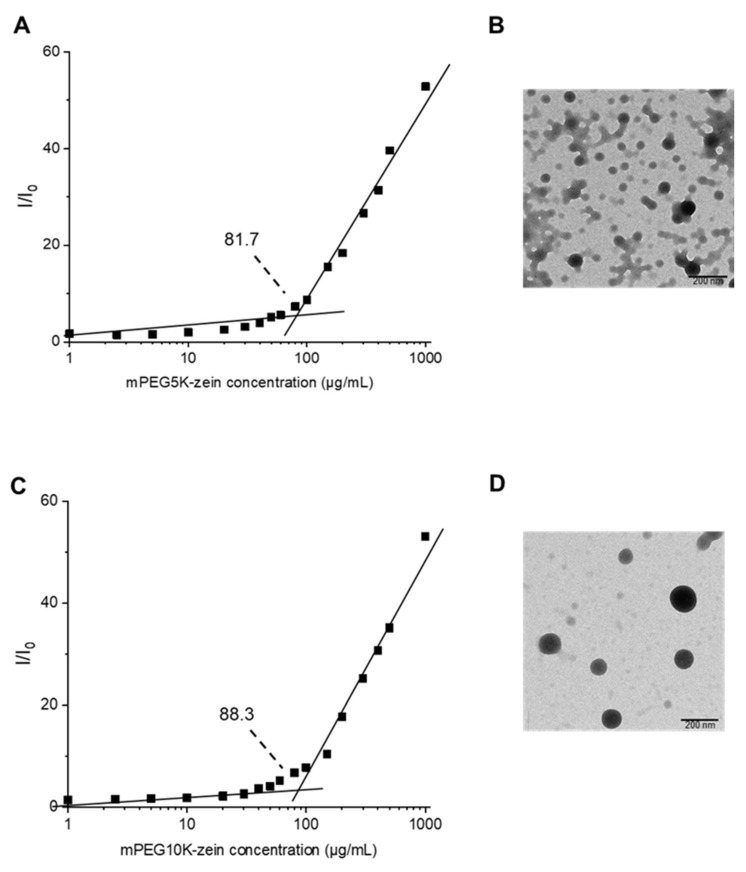
Assembly of mPEG-zein into micelles: relative fluorescence intensity (I/I_o_) of Nile red in the function of the concentration of mPEG5K-zein (**A**) and mPEG10K-zein (**C**), unstained TEM images of mPEG5K-zein (**B**) and mPEG10K-zein micelles (**D**) (scale bar: 200 nm).

The assembly behavior of mPEG-zein was further studied by a steady-state fluorescence technique using Nile red as a hydrophobic fluorescent probe. Nile red is weakly fluorescent in water. However, its fluorescence intensity increased with increasing mPEG-zein concentrations, as Nile red was solubilized within the hydrophobic core of the micelles [29]. The CMC values decreased by reducing the MW of conjugated PEG (81.7 µg/mL for PEG5K versus 88.3 µg/mL for PEG10K) (Figure 2A,C). This could be explained by the overall higher hydrophobic content of the smaller PEG conjugate over the larger PEG conjugate, as the micelle formation occurs mainly through the hydrophobic interactions among zein molecules. The CMC value obtained for mPEG5K-zein micelles was relatively higher than that previously published (55 µg/mL and 20 µg/mL in Podaralla et al. [19] and Song et al. [20], respectively), when using pyrene as a fluorescence probe. This difference might be due to the different hydrophobic probes used, as well as the type of zein used as starting material. Podaralla et al. used white zein, and Song et al. used purified α-zein, while yellow zein was chosen in our study [19,20]. Chan et al. previously demonstrated that the CMC value for diblock copolymer micelles characterized using pyrene was lower than that using Nile red (15.1 mg/mL by pyrene versus 19.5 mg/mL by Nile red) [30]. Nevertheless, the CMC values obtained in our study were low, indicating high stability of the mPEG-zein micelles.

TEM images revealed that the obtained micelles had a spherical shape (Figure 2B,D). The size of mPEG5K-zein micelles appeared to be smaller than that of their 10 K counterpart. This was further confirmed by DLS measurements. mPEG5K-zein and mPEG10K-zein micelles displayed particle diameters of 147.1 ± 3.5 nm and 280.1 ± 42.9 nm, with PDI values of 0.2 ± 0.1 and 0.4 ± 0.1, respectively. They were larger than the corresponding sizes measured by TEM, as TEM imaging involves drying of the samples before measurement, unlike DLS, where hydrodynamic sizes are measured. However, these micelles should achieve passive accumulation within solid tumors due to the enhanced permeability and retention (EPR) effect, as their sizes were smaller than the cut-off size for extravasation (400 nm) [31].

Both types of micelles loaded with Nile red bore similar positive zeta potential (24.3 ± 1.9 mV for mPEG5K-zein and 22.7 ± 0.4 mV for mPEG10K-zein), suggesting that the MW of PEG used for the conjugation had no substantial effect on the particle surface charge. The entrapment of Nile red did not alter the surface charge of the micelles, as similar zeta potential values were observed for empty mPEG-zein micelles (25.6 ± 1.2 mV for mPEG5K-zein and 23.6 ± 2.5 mV for mPEG10K-zein), thus further confirming that Nile red was entrapped in the inner core of the micelles. The positive net surface charge of mPEG5K-zein micelles was in agreement with that presented by Song et al. when using α-zein in their micelle formulation (39 mV) [20]. It would increase the electrostatic interactions between the micelles and the negatively charged cell membrane, resulting in an increased cellular uptake of the delivery system [29].

The modulation of the MW of PEG conjugated to zein had an impact on the entrapment of Nile red in the micelles, which increased when using larger MW PEG (respectively 70.5 ± 3.8% (loading content: 3.5 ± 0.2 µg of Nile red per mg of mPEG-zein) and 84.7 ± 2.9% (loading content: 4.2 ± 0.1 µg of Nile red per mg of mPEG-zein) for mPEG5K-zein and mPEG10K-zein micelles). The increased entrapment with the higher PEG MW was also observed in PEG-coated fluconazole nanoparticles [32]. However, in some delivery systems, such as PEG-PLGA and chitosan nanoparticles, the encapsulation efficiency was independent of the PEG MW [33,34]. It was even found to decrease with increasing PEG MW, for example, in the case of cholesterol-bearing PEGylated polymeric micelles, due to the overall low cholesterol content in the co-polymer chain for polymers with higher MW PEG, making these micelles less favorable for drug encapsulation [35].

### 3.3. Cellular Uptake of mPEG-Zein Micelles

**Figure 3:**
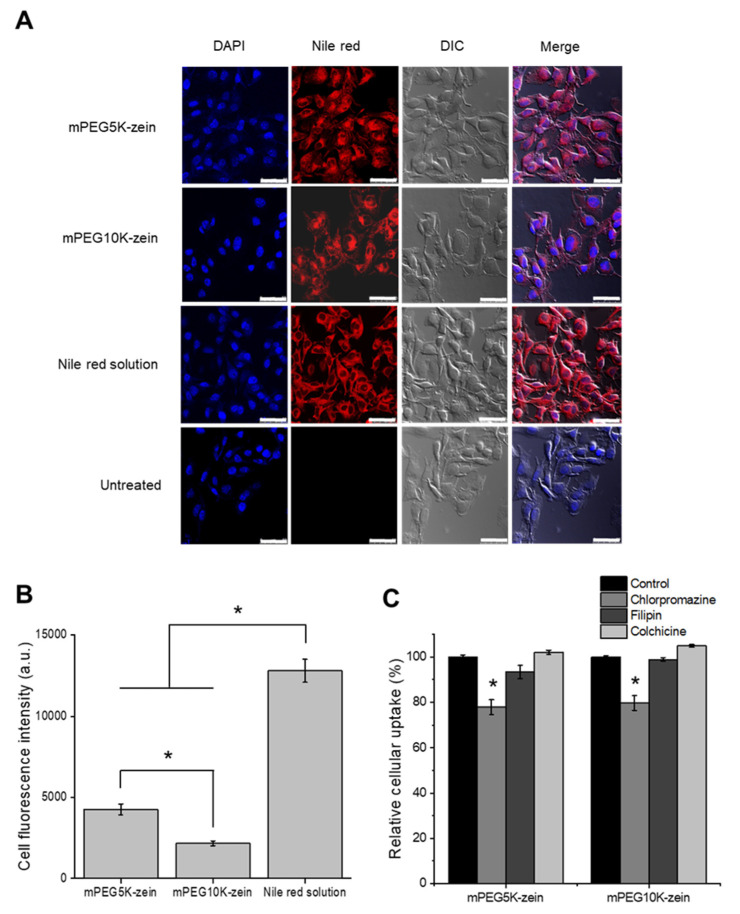
Uptake of mPEG-zein micelles by B16-F10-luc-G5 cells. (**A**) Confocal images of B16-F10-luc-G5 cells after incubation with Nile red loaded in mPEG-zein micelles, or as a solution (DIC: differential interference contrast), (scale bar: 50 µm). (**B**) Flow cytometry analysis of the cellular uptake of Nile red loaded in mPEG-zein micelles or as a solution (a.u.: arbitrary units) (*n* = 3) (*: *p* < 0.05). (**C**) Effects of endocytosis inhibitors on the cellular uptake of Nile red-loaded mPEG-zein micelles (*n* = 3) (*: *p* < 0.05, compared with control).

The uptake of mPEG-zein micelles by B16-F10-luc-G5 melanoma cancer cells was qualitatively evaluated using confocal microscopy (Figure 3A). Both mPEG-zein micelles could deliver Nile red into the cells following 2 h incubation. Fluorescent Nile red, predominantly located in the cytoplasm, was also found to be co-localized in the nuclei following treatment with mPEG5K-zein micelles, unlike mPEG10K-zein, which showed weak red fluorescence signals within the nucleus. Higher Nile red accumulation in the cells was observed from Nile red solution over the micelles.

The cellular uptake of mPEG-zein micelles was further confirmed by flow cytometry (Figure 3B and Figure S2). The mean fluorescence intensity (MFI) of cells incubated with Nile red solution (12,796 ± 712 arbitrary units (a.u.)) was at least three-fold higher than that of mPEG-zein micelles, which correlated well with the observation from confocal microscopy. This could be explained by the different cellular uptake mechanisms used by Nile red solution and the micelles: passive diffusion of the Nile red solution to the cells, while the micelles are taken up by endocytosis. Furthermore, mPEG5K-zein micelles were more efficacious in delivering Nile red into the cells, by approximately two-fold in comparison with its longer chain counterparts (MFI of 4256 ± 195 a.u. for mPEG5K-zein versus MFI of 2169 ± 85 a.u. for mPEG10K-zein), therefore demonstrating that PEGylation with a shorter PEG chain length could improve the cellular uptake efficacy of the zein micelles. Higher uptake with a shorter PEG chain length was consistent with several types of PEGylated nanocarriers [34,36,37]. Increasing PEG MW was shown to prevent nanoparticle-cell interactions, as higher MW PEG grafting led to a larger surface shielding of the micelles [38,39]. Hence, a relatively lower uptake of the mPEG10K formulation compared to the mPEG5K-based one in cancer cells could be explained by a decrease in cell adhesion.

To investigate the endocytosis pathways of mPEG-zein micelles entering B16-F10-luc-G5 cells, various pathway-specific inhibitors were used: chlorpromazine as the inhibitor of clathrin-mediated endocytosis, filipin as the inhibitor of caveolae-mediated endocytosis, and colchicine as the inhibitor of macropinocytosis [40]. The uptake of both mPEG-zein micelles was inhibited by chlorpromazine by about 20% (Figure 3C), indicating that these micelles were mainly internalized by clathrin-mediated endocytosis, which is the main route by which nanocarriers enter the cells [40]. Filipin and colchicine did not decrease the cellular uptake of the micelles, suggesting that caveolae-mediated and macropinocytosis-mediated endocytosis did not participate in the uptake of the micelles. The cellular uptake of other zein-based delivery systems via energy-dependent endocytosis was also previously reported, however involving different endocytosis pathways. Doxorubicin-loaded zein nanoparticles and caseinate-zein nanoparticles involved macropinocytosis instead of caveolin-mediated or clathrin-mediated pathways [16,41].

### 3.4. Size and Zeta Potential of mPEG-Zein Micelles in the Presence of Proteins

mPEG5K-zein and mPEG10K-zein micelles were incubated in cell culture medium in the presence of proteins, and their size were measured at various times by DLS. The micelle size slightly increased in the presence of FBS (Figure S3), probably due to the formation of the protein corona on the surface of the micelles, as previously reported [42,43,44,45]. However, the magnitude of the size change can vary depending on the delivery systems. For example, the average size of black phosphorus nanosheets-corona complexes increased by 8% (from 338.4 ± 2.3 nm to 365.3 ± 5.9 nm), while the diameter of black phosphorus quantum dots unexpectedly increased over 6000% (from 5.6 ± 1.4 nm to 362.5 ± 5.6 nm) after the protein corona was formed [45].

**Figure 4:**
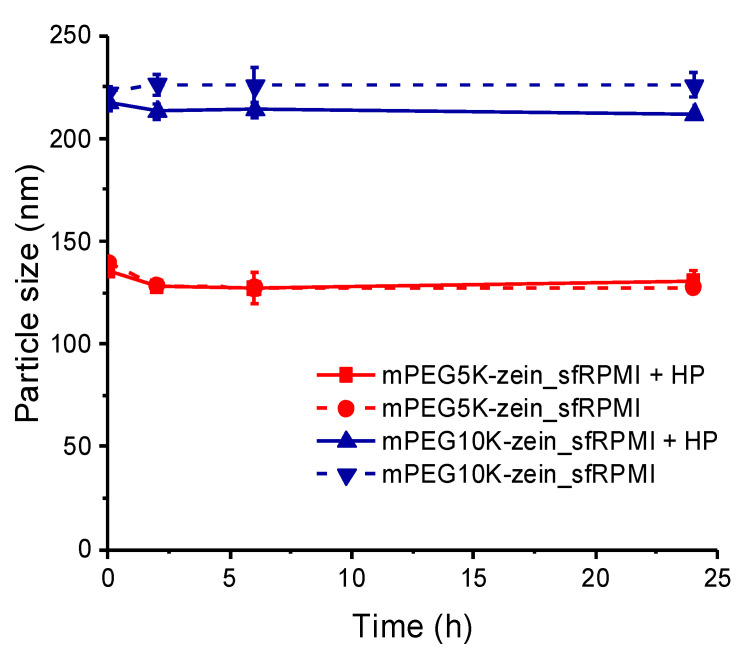
Size of mPEG5K-zein and mPEG10K-zein micelles in the presence or absence of human plasma (HP) (“sfRPMI”: serum-free RPMI medium, “sfRPMI + HP”: serum-free RPMI medium + 10% (*v*/*v*) HP) (*n* = 3).

When the micelles were allowed to interact with HP, such an increase in size was not observed (Figure 4). The size of mPEG5K-zein micelles stayed the same over 24 h, independent of the presence or absence of HP (from 135.4 ± 1.4 nm to 130.9 ± 4.5 nm after 24 h). Likewise, mPEG10K-zein micelles displayed a similar size over 24 h. The presence of HP led to a slight decrease of the micelle size (212.1 ± 0.9 nm and 225.9 ± 6.0 nm, respectively with and without HP after 24-h incubation). In general, proteins interact with nanoparticles by forming a corona around their surface, resulting in a thickening of the nanoparticle surface and a subsequent increase in their size [42,43,44,45]. However, they may lead to a size reduction due to osmotically driven shrinkage [46]. For our delivery systems, the latter effect may be predominant, particularly for the mPEG10K formulation. Repulsive forces of mPEG10K-zein micelles against HP could prevent the formation of a dense protein corona. Instead, proteins that are impermeable to the micelles might induce osmotic pressure, which caused water to escape from the micelle and the micelle to shrink.

Although these micelles did not exhibit an increase in their size following incubation with HP, a drop in zeta potential values by about 8 mV was observed (from 28.6 ± 1.0 mV to 20.3 ± 2.7 mV for mPEG5K-zein and from 30.9 ± 0.4 mV to 23.0 ± 0.8 mV for mPEG10K-zein). This might be due to the binding of negatively charged proteins on the micelle surface. The neutralization of the particle surface charge resulting from the binding of proteins with opposite charges on the nanoparticle surface was in accordance with data published by several groups for other delivery systems [37,45,47].

Surface modification of delivery systems with PEG has been reported to prevent nonspecific interactions with proteins as hydrophilic PEG chains become compressed when proteins approach the surface, thus creating a thermodynamic barrier to protein adsorption [25,48,49,50]. The presence of FBS or HP in the medium surrounding the micelles led to minimal changes in the hydrodynamic radius, indicating limited micelle–protein interactions. Our results suggest that both mPEG5K-zein and mPEG10K-zein micelles exhibited stealth properties regardless of the PEG chain length.

### 3.5. Effect of Protein Corona on the Cellular Uptake of mPEG-Zein Micelles

**Figure 5:**
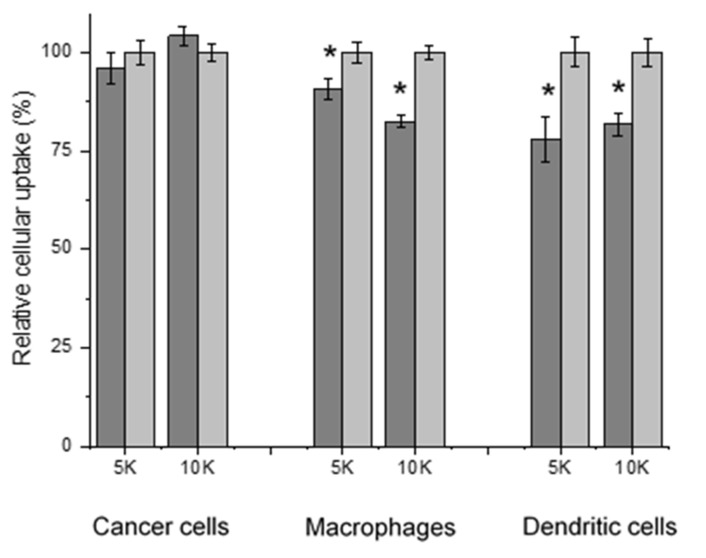
Cellular uptake of Nile red loaded in mPEG5K-zein (“5K”) and mPEG10K-zein (“10K”) micelles precoated with HP (gray) or glucose 5% (*w*/*v*) solution as control (light gray), by B16-F10-luc-G5 cancer cells, macrophages, and dendritic cells (*n* = 6) (*: *p* < 0.05, compared with controls without protein corona).

To investigate the impact of protein corona on drug delivery vehicles in contact with biological fluids, HP was used to prepare pre-formed corona micelles. In this study, Nile red-loaded mPEG5K-zein and mPEG10K-zein micelles were pre-incubated in HP or in a glucose solution (5%, *w*/*v*) at 37 °C for 1 h to allow protein adsorption on the surface of the micelles. They were then added to B16-F10-luc-G5 cells in a serum-free medium for 2 h to determine the effect of a preformed corona on cellular uptake levels. Flow cytometry analysis revealed that pre-coating the micelles with HP had no substantial effect on the uptake of Nile red by the cancer cells, independent of the PEG chain length used in the formulations (Figure 5).

However, following intravenous administration, nanomaterials are often covered by corona proteins. Some of them can act as opsonins to influence the recognition and clearance of the particles by cells of the mononuclear phagocyte system (MPS), predominantly dendritic cells in the bloodstream and macrophages at tissues, and thus potentially prevent the particles from reaching their target tumors [45,48]. Therefore, the cellular uptake of the preformed corona mPEG-zein micelles by murine bone marrow-derived macrophages and dendritic cells was also evaluated. As shown in Figure 5, precoating the micelles with HP resulted in a decreased uptake by macrophages (9% uptake reduction for mPEG5K-zein and 18% uptake reduction for its mPEG10K counterpart). The uptake of mPEG5K-zein and mPEG10K-zein micelles by dendritic cells was also reduced by 22% and 19%, respectively after pre-incubation with HP. The adsorption of HP on the micelle surface, therefore, had a positive impact on the uptake of the formulations by macrophages and dendritic cells. The effect of FBS on the cellular uptake of mPEG-zein micelles was also studied. We found that the presence of FBS slightly decreased the uptake of the micelles by all three cell types (Figure S4). The small effect of the protein corona on the cellular uptake of our mPEG-zein micelles could be explained by a decreased protein binding resulting from the stealth properties of PEG [51].

The decrease in the cellular uptake of pretreated micelles (human plasma) by macrophages and dendritic cells could be explained by the lowered micelle–cell membrane adhesion caused by the adsorption of proteins around the micelle surface. The small decrease in cellular uptake is probably due to the decreased protein binding resulting from the stealth properties of PEG. Furthermore, PEGylation could not inhibit serum protein adsorption completely, even at high grafting density, but it could selectively suppress the adsorption of specific proteins, such as opsonins [48]. Taken together, these two factors might be the reason for a decreased uptake by macrophages and dendritic cells. This stealth effect is not specific to mPEG, as Schöttler et al. [52] also observed a decrease in protein adsorption and the presence of a specific protein that was necessary to prevent a nonspecific cellular uptake of polystyrene nanocarriers after modification with poly(ethyl ethylene phosphate) (PEEP).

The relationship between the protein corona and nanoparticle uptake efficiency has previously been investigated in several nanomaterials. Lesniak et al., for instance, demonstrated a significant inhibition of the uptake of silica nanoparticles in the presence of serum by human lung cancer A549 cells (1,500,000 a.u. in serum-free versus 10,500 a.u. in complete medium, after 2 h incubation) [43]. As nanoparticle uptake involves cell membrane adsorption followed by subsequent internalization via energy-dependent endocytosis [53], the lowered particle–cell membrane adhesion due to the adsorption of serum proteins on the nanoparticle surface thereby caused a reduction in internalization efficiency [54]. Nevertheless, a protein corona could affect nanoparticle uptake efficiency with different outcomes. Yan et al. reported that the adsorption of FBS on disulfide-stabilized poly-(methacrylic acid) nanoporous polymer particles significantly decreased the cellular uptake in monocytes by at least 50%. In contrast, it did not affect the uptake level in macrophages [44]. Pozzi et al. also demonstrated that the cell internalization of PEGylated multicomponent cationic liposomes in PC3 prostate cancer cells decreased from 80% to 50% after incubation with HP, but the cellular uptake of non-PEGylated liposome–HP complexes increased with respect to their counterparts in the absence of the corona [37].

The viability of B16-F10-luc-G5 cancer cells, macrophages, and dendritic cells was assessed following treatment with Nile red-loaded mPEG-zein micelles at the experimental conditions used in the cellular uptake experiments (same concentration and treatment for 4 h for the cancer cells and 2 h for the macrophages and dendritic cells). The viability of the three cell types was higher than 80% following treatment with both micelle formulations, suggesting that the micelles were not toxic to the cells at these experimental conditions. It was not significantly different from that observed with cells treated with Nile red solution or untreated cells (Figure S5). This confirmed that the differences in the cellular uptake analysis were not associated with the toxicity of the micelles.

### 3.6. Analysis of the Protein Corona

The hard corona proteins were obtained after 1 h incubation of mPEG-zein micelles in HP, followed by two-step centrifugation. They were then separated using SDS-PAGE and visualized by silver staining to gain an overview about their protein signatures. The protein patterns of mPEG5K-zein and mPEG10K-zein micelles were almost identical (Figure S6). Both micelles were mainly covered by albumin (band at ~62 kDa), the protein with the highest concentration in blood [44,55]. However, it is well accepted that numerous proteins that exhibit a low abundance in blood are also highly enriched on the particle surface [50,52]. Therefore, nLC-ESI–MS/MS was applied for the identification and the relative quantification of the hard corona composition, which allowed for more detailed insights about the protein adsorption behavior.

**Figure 6:**
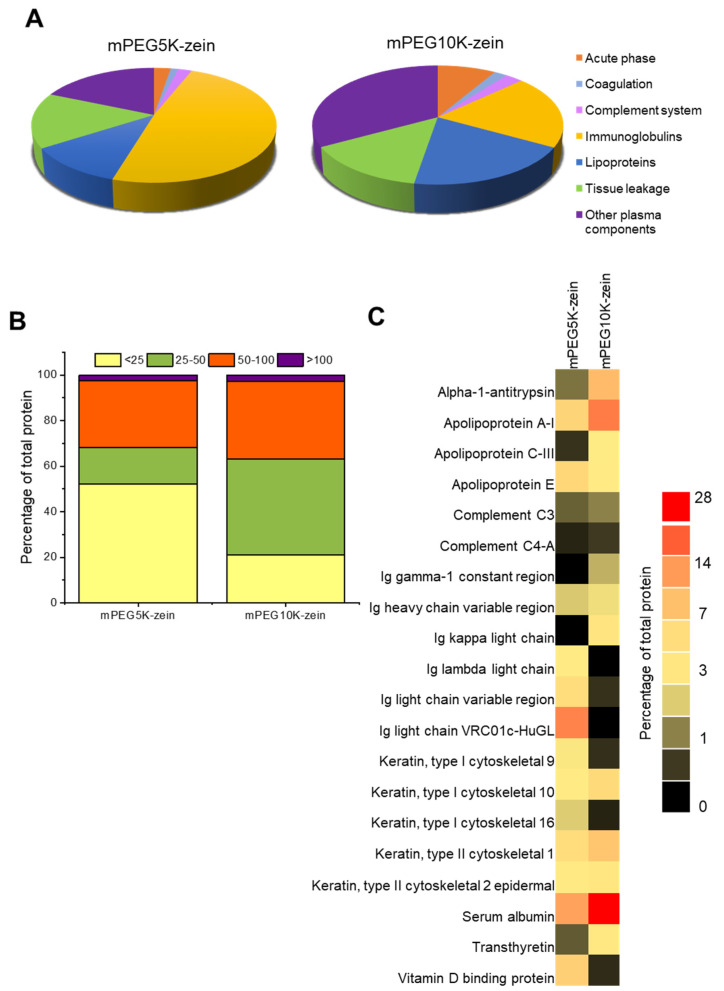
Proteomic analysis of the protein corona surrounding mPEG5K-zein and mPEG10K-zein micelles. Proteins were classified by function (**A**) and weight (kDa) (**B**). The 20 most abundant proteins in the corona of mPEG-zein micelles were used to create the heat map (**C**).

A total of 132 and 109 proteins were identified from the protein coronas recovered from mPEG5K-zein and mPEG10K-zein samples, respectively. Of these proteins, 48 proteins appeared on both types of micelles. All identified proteins of varying MW, molecular weight search (MOWSE) score, and exponentially modified protein abundance index (emPAI) are presented in Table S1. Grouping the proteins according to their MW showed that low MW proteins (<25 kDa) were predominantly found on mPEG5K-zein micelles, while proteins with medium size (25–100 kDa) were significantly enriched on mPEG10K-zein micelles (Figure 6B). We also detected a very low level of high MW proteins (>100 kDa) on the surface of both micelles. This result indicates a distinct protein binding pattern on each micelle type.

The bound proteins were further classified according to their functions (Figure 6A). Both micelle types were mainly covered by immunoglobulins, lipoproteins, the proteins involved in tissue leakage, and other plasma components. We found a significant enrichment of immunoglobulins (49%) on the surface of mPEG5K-zein micelles. The most abundant protein from the corona recovered from mPEG5K-zein micelles was the immunoglobulin light chain (13%), followed by albumin (10%). In contrast, proteins that preferentially bound to mPEG10K-zein micelles were other plasma components (33%). Lipoproteins and immunoglobulins constituted up to 40% of the protein corona (~20% from each group). The highest enrichment of proteins on the surface of mPEG10K-zein micelles was albumin (27%), and the next most abundant protein was apolipoprotein A-I (14%) (Figure 6C).

Our results suggest that plasma protein adsorption was influenced by PEG MW, since different protein compositions and contents were detected from the corona of both micelle types. These results were supported by several publications. Gref et al. revealed that the type and amount of the corona proteins were determined by the PEG chain length and density at the particle surface as well as the nature of the core material [25]. In a recent study, Partikel et al. observed a significant depletion of bound proteins, opsonins, in terms of the amount and number, due to PEGylation on PLGA nanoparticles [50]. The adsorption of opsonins, such as immunoglobulins and complement factors, onto the nanoparticle surface is thought to promote phagocytosis and clearance of the particles by cells of the MPS. By contrast, the binding of dysopsonins, such as albumin, has been shown to prolong blood circulation lifetime. Ogawara et al. reported that pre-coating polystyrene nanoparticles with albumin could suppress the association of serum proteins with opsonic activity, resulting in prolonged blood circulation after intravenous injection in rats [56]. Instead of albumin, Schöttler et al. identified clusterin (also known as apolipoprotein J) as another protein with dysopsonic properties [52]. Clusterin was also detected in our hard corona proteins, but at very low levels (<1% in both micelle systems). However, we found albumin and various apolipoproteins, mainly apolipoproteins A-I and E, to be prominent in the protein corona recovered from both micelles. Apolipoproteins generally exhibit dysopsonic function, as reported previously [50,52]. In summary, even though immune relevant proteins, such as immunoglobulins, were present in high percentages in the hard corona recovered from our micelles, in particular in the case of PEG5K, we also found an enrichment of dysopsonins, such as albumin and apolipoproteins. The presence of dysopsonins might antagonize the biological effects of micelle-bound opsonins [55], leading to a limited decrease in cellular uptake by macrophages and dendritic cells.

## 4. Conclusions

In this work, we demonstrated that yellow zein could be successfully conjugated with PEG and was able to assemble into micelles, entrapping a model hydrophobic substance, Nile red. mPEG-zein micelles could deliver Nile red into the B16-F10-luc-G5 melanoma cell line via clathrin-mediated endocytosis, with a higher cellular uptake observed when using smaller chain length PEG5K. The present study provides the first investigation of the impact of the protein corona on mPEG-zein micelle uptake by cancer cells and immune cells. Overall, PEGylation of zein could confer stealth effects on the micelle surface, regardless of the PEG chain length, thereby minimizing the adsorption of proteins on the micelles. Most importantly, it was shown for the first time that the presence of HP did not have any impact on the uptake of mPEG-zein micelles by the melanoma cancer cells, independent of the MW PEG used in the formulations. In addition, it decreased the uptake of the micelles by macrophages and by dendritic cells for both micelle formulations. This effect might be due to the presence of dysopsonins, such as albumin and apolipoproteins, in the hard corona surrounding the micelles that might antagonize the biological effects of the micelle-bound opsonins. These results, therefore, make PEGylated zein micelles promising candidates to further investigate as drug delivery vehicles for cancer therapy.

## Figures and Tables

**Table 1:** Composition of the two mPEG-zein synthesized in the study.

	Composition	Weight Ratio	Molar Ratio
mPEG5K-zein	mPEG5K:zein	0.5:1	2.3:1
mPEG10K-zein	mPEG10K:zein	1:1	2.3:1

## Data Availability

The data that support the findings of this study are available from the corresponding author, [C.D.], upon request.

## Supplementary Materials

The following supporting information can be downloaded at: https://www.mdpi.com/article/10.3390/pharmaceutics14020439/s1, Figure S1. FTIR spectra of zein (A), mPEG5K (B), mPEG10K (C), mPEG5K-zein conjugate (D), mPEG10K-zein conjugate (E), non-conjugated mixture of mPEG5K with zein (F), and non-conjugated mixture of mPEG10K with zein (G); Figure S2. Flow cytometry histograms of B16-F10-luc-G5 cells following 2 h incubation with Nile red loaded in mPEG5K-zein (A) and mPEG10K-zein (B) micelles, Nile red solution (C), or left untreated (D); Figure S3. Size of mPEG5K-zein and mPEG10K-zein micelles in the presence or absence of 10% (*v*/*v*) FBS, over 24 h (“cDMEM”: complete DMEM medium, “sfDMEM”: serum-free DMEM medium) (*n* = 3); Figure S4. Effect of FBS on the cellular uptake of mPEG-zein micelles. Nile red-loaded mPEG5K-zein and mPEG10K-zein micelles were pre-incubated in complete medium or in serum-free medium at 37°C for 1 h to allow protein adsorption to the surface of the micelles. (A) Time-dependent uptake of the pre-formed corona micelles in complete and serum-free medium by B16-F10-luc-G5 cells (cRPMI: complete RPMI medium, sfRPMI: serum-free RPMI medium) (*n* = 3). (B) Cellular uptake of the pre-formed corona micelles in cRPMI (dark gray) or sfRPMI (light gray) by macrophages and dendritic cells (*n* = 6) (*: *p* < 0.05, compared with sfRPMI); Figure S5. Viability of B16-F10-luc-G5 cancer cells (A), macrophages (B), and dendritic cells (C) treated with Nile red-loaded mPEG-zein micelles for 4 h (*n* = 15) (cancer cells) or 2 h (*n* = 10) (macrophages and dendritic cells) (controls: cells treated with Nile red solution or left untreated). There was no statistical difference between the treatments; Figure S6. SDS-PAGE gels of protein corona surrounding mPEG-zein micelles following incubation in cDMEM, sfDMEM (A) and HP (B) at 37 °C for 1 h. The analysis was performed in duplicate—for clarity only one replicate is shown. Protein bands at ~22–24 kDa correspond to α-zein. Other zein fractions were also detected from micelles that were incubated with sfDMEM. However, the intensities of these bands were considerably low; Table S1. List of hard corona proteins on mPEG5K-zein and mPEG10K-zein micelles after exposure to human plasma at 37 °C for 1 h (n.d.: not detected).

## References

[1] Lawton J.W. (2002). Zein: A history of processing and use. Cereal Chem..

[2] Luo Y., Zhang B., Cheng W.H., Wang Q. (2010). Preparation, characterization and evaluation of selenite-loaded chitosan/TPP nanoparticles with or without zein coating. Carbohyd. Polym..

[3] Lin T., Lu C., Zhu L., Lu T. (2011). The biodegradation of zein *in vitro* and *in vivo* and its application in implants. AAPS.

[4] Shukla R., Cheryan M. (2001). Zein: The industrial protein from corn. Ind. Crops Prod..

[5] Phillips R.L., McClure B.A. (1985). Elevated protein-bound methionine in seeds of maize line resistant to lysine plus threonine. Cereal Chem..

[6] Parris N., Dickey L.C. (2001). Extraction and solubility characteristics of zein proteins from dry-milled corn. J. Agric. Food Chem..

[7] Podaralla S., Perumal O. (2012). Influence of formulation factors on the preparation of zein nanoparticles. AAPS PharmSciTech.

[8] Paliwal R., Palakurthi S. (2014). Zein in controlled drug delivery and tissue engineering. J. Control. Release.

[9] Gianazza E., Viglienghi V., Righetti P.G., Salamini F., Soave C. (1977). Amino acid composition of zein molecular components. Phytochemistry.

[10] Wang H.J., Gong S.J., Lin Z.X., Fu J.X., Xue S.T., Huang J.C., Wang J.Y. (2007). *In vivo* biocompatibility and mechanical properties of porous zein scaffolds. Biomaterials.

[11] Gong S.J., Sun S.X., Sun Q.S., Wang J.Y., Liu X.M., Liu G.Y. (2011). Tablets based on compressed zein microspheres for sustained oral administration: Design, pharmacokinetics, and clinical study. J. Biomater. Appl..

[12] Demir M., Romos-Rivera L., Silva R., Nazhat S.N., Boccaccini A.R. (2017). Zein-based composites in biomedical applications. J. Biomed. Mater. Res. A.

[13] Liu X., Sun Q., Wang H., Zhang L., Wang J.Y. (2005). Microspheres of corn protein, zein, for an ivermectin drug delivery system. Biomaterials.

[14] Parris N., Cooke P.H., Hicks K.B. (2005). Encapsulation of essential oils in zein nanospherical particles. J. Agric. Food Chem..

[15] Fu J.X., Wang H.J., Zhou Y.Q., Wang J.Y. (2009). Antibacterial activity of ciprofloxacin-loaded zein microsphere films. Mater. Sci. Eng. C.

[16] Dong F., Dong X., Zhou L., Xiao H., Ho P.Y., Wong M.S., Wang Y. (2016). Doxorubicin-loaded biodegradable self-assembly zein nanoparticle and its anti-cancer effect: Preparation, *in vitro* evaluation, and cellular uptake. Colloids Surf. B Biointerfaces.

[17] Thapa R.K., Nguyen H.T., Jeong J.H., Shin B.S., Ku S.K., Choi H.G., Yong C.S., Kim J.O. (2017). Synergistic anticancer activity of combined histone deacytylase and proteasomal inhibitor-loaded zein nanoparticles in metastatic prostate cancers. Nanomed. NBM.

[18] Hurtado-Lopez P., Murdan S. (2006). Zein microspheres as drug/antigen carriers: A study of their degradation and erosion, in the presence and absence of enzymes. J. Microencapsul..

[19] Podaralla S., Averineni R., Alqahtani M., Perumal O. (2012). Synthesis of novel biodegradable methoxy poly(ethylene glycol)–zein micelles for effective delivery of curcumin. Mol. Pharm..

[20] Song R., Zhou Y., Li Y., Yang Z., Li F., Huang Q., Shi T., Zhang G. (2015). Preparation and characterization of mPEG-g-α-zein biohybrid micelles as a nano-carrier. J. Appl. Polym. Sci..

[21] Albanese A., Tang P.S., Chan W.C. (2012). The effect of nanoparticle size, shape, and surface chemistry on biological systems. Annu. Rev. Biomed. Eng..

[22] Corbo C., Molinaro R., Porodi A., Toledano Furman N.E., Salvatore F., Tasciotti E. (2016). The impact of nanoparticle protein corona on cytotoxicity, immunotoxicity and target drug delivery. Nanomedicine.

[23] Nguyen V.H., Lee B.J. (2017). Protein corona: A new approach for nanomedicine design. Int. J. Pharm..

[24] Monopoli M.P., Aberg C., Salvati A., Dawson K.A. (2012). Biomolecular coronas provide the biological identity of nanosized materials. Nat. Nanotechnol..

[25] Gref R., Lück M., Quellec P., Marchand M., Dellacherie E., Harnisch S., Blunk T., Müller R.H. (2000). ‘Stealth’ corona-core nanoparticles surface modified by polyethylene glycol (PEG): Influences of the corona (PEG chain length and surface density) and of the core composition on phagocytic uptake and plasma protein adsorption. Colloids Surf. B Biointerfaces.

[26] Owens D.E., Peppas N.A. (2006). Opsonization, biodistribution, and pharmacokinetics of polymeric nanoparticles. Int. J. Pharm..

[27] Schroeder J., McGachy H.A., Woods S., Plevin R., Alexander J. (2013). T Cell Hypo-Responsiveness against *Leishmania major* in MAP Kinase Phosphatase (MKP) 2 Deficient C57BL/6 Mice Does Not Alter the Healer Disease Phenotype. PLoS Negl. Trop. Dis..

[28] He W., Tian L., Fang F., Chen D., Federici E., Pan S., Jones O.G. (2021). Limited hydrolysis and conjugation of zein with chitosan oligosaccharide. Food Chem..

[29] Laskar P., Somani S., Altwaijry N., Mullin M., Bowering D., Warzecha M., Keating P., Tate R.J., Leung H.Y., Dufès C. (2018). Redox-sensitive, cholesterol-bearing PEGylated poly(propylene imine)-based dendrimersomes for drug and gene delivery to cancer cells. Nanoscale.

[30] Chan D., Yu A.C., Appel E.A. (2017). Single-chain polymeric nanocarriers: A platform for determining structure-function correlations in the delivery of molecular cargo. Biomacromolecules.

[31] Yuan F., Dellian M., Fukumura D., Leunig M., Berk D.A., Torchilin V.P., Jain R.K. (1995). Vascular permeability in a human tumor xenograft: Molecular size dependence and cutoff size. Cancer Res..

[32] Abdellatif A.A.H., El-Telbany D.F.A., Zayed G., Al-Sawahli M.M. (2019). Hydrogel containing PEG-coated fluconazole nanoparticles with enhanced solubility and antifungal activity. J. Pharm. Innov..

[33] Cruz L.J., Tacken P.J., Fokkink R., Figdor C.G. (2011). The influence of PEG chain length and targeting moiety on antibody-mediated delivery of nanoparticle vaccines to human dendritic cells. Biomaterials.

[34] Bachir Z.A., Huang Y., He M., Huang L., Hou X., Chen R., Gao F. (2018). Effect of PEG surface density and chain length on the pharmacokinetics and biodistribution of methotrexate-loaded chitosan nanoparticles. Int. J. Nanomed..

[35] Laskar P., Samanta S., Ghosh S.K., Dey J. (2014). In vitro evaluation of pH-sensitive cholesterol-containing stable polymeric micelles for delivery of camptothecin. J. Colloid. Interface Sci..

[36] Cruje C., Chithrani D.B. (2014). Polyethylene glycol density and length affects nanoparticle uptake by cancer cells. J. Nanomed. Res..

[37] Pozzi D., Colapicchioni V., Caracciolo G., Piovesana S., Capriotti A.L., Palchetti S., de Grossi S., Riccioli A., Amenitsch H., Laganà A. (2014). Effect of polyethyleneglycol (PEG) chain length on the bio-nano-interactions between PEGylated lipid nanoparticles and biological fluids: From nanostructure to uptake in cancer cells. Nanoscale.

[38] Du H., Chandaroy P., Hui S.W. (1997). Grafted poly(ethylene glycol) on lipid surfaces inhibits protein adsorption and cell adhesion. Biochim. Biophys. Acta.

[39] Suk J.S., Xu Q., Kim N., Hanes J., Ensign L.M. (2016). PEGylation as a strategy for improving nanoparticle-based drug and gene delivery. Adv. Drug Deliv. Rev..

[40] Cheng L., Huang F.Z., Cheng L.F., Zhu Y.Q., Hu Q., Li L., Wei L., Chen D.-W. (2014). GE11-modified liposomes for non-small cell lung cancer targeting: Preparation, *ex vitro* and *in vivo* evaluation. Int. J. Nanomed..

[41] Luo Y., Teng Z., Wang T.T.Y., Wang Q. (2013). Cellular uptake and transport of zein nanoparticles: Effects of sodium caseinate. J. Agric. Food Chem..

[42] Shapero K., Fenaroli F., Lynch I., Cottell D.C., Salvati A., Dawson K.A. (2011). Time and space resolved uptake study of silica nanoparticles by human cells. Mol. Biosyst..

[43] Lesniak A., Fenaroli F., Monopoli M.P., Aberg C., Dawson K.A., Salvati A. (2012). Effect of the presence or absence of a protein corona on silica nanoparticle uptake and impact on cells. ACS Nano.

[44] Yan Y., Gause K.T., Kamphuis M.M.J., Ang C.S., O’Brien-Simpson N.M., Lenzo J.C., Reynolds E.C., Nice E.C., Caruso F. (2013). Differential roles of the protein corona in the cellular uptake of nanoporous polymer particles by monocyte and macrophage cell lines. ACS Nano.

[45] Mo J., Xie Q., Wei W., Zhao J. (2018). Revealing the immune perturbation of black phosphorus nanomaterials to macrophages by understanding the protein corona. Nat. Commun..

[46] Wolfram J., Suri K., Yang Y., Shen J., Celia C., Fresta M., Zhao Y., Shen H., Ferrari M. (2014). Shrinkage of pegylated and non-pegylated liposomes in serum. Colloids Surf. B Biointerfaces.

[47] Gräfe C., Weidner A., Lühe M.V.D., Bergemann C., Schacher F.H., Clement J.H., Dutz S. (2016). Intentional formation of a protein corona on nanoparticles: Serum concentration affects protein corona mass, surface charge, and nanoparticle-cell interaction. Int. J. Biochem. Cell. Biol..

[48] Walkey C.D., Olsen J.B., Guo H., Emili A., Chan W.C.W. (2012). Nanoparticle size and surface chemistry determine serum protein adsorption and macrophage uptake. J. Am. Chem. Soc..

[49] Dai Q., Walkey C., Chan W.C.W. (2014). Polyethylene glycol backfilling mitigates the negative impact of the protein corona on nanoparticle cell targeting. Angew. Chem. Int. Ed..

[50] Partikel K., Korte R., Stein N.C., Mulac D., Herrmann F.C., Humpf H.U., Langer K. (2019). Effect of nanoparticle size and PEGylation on the protein corona of PLGA nanoparticles. Eur. J. Pharm. Biopharm..

[51] Otsuka H., Nagasaki Y., Kataoka K. (2012). PEGylated nanoparticles for biological and pharmaceutical applications. Adv. Drug Deliv. Rev..

[52] Schöttler S., Becker G., Winzen S., Steinbach T., Mohr K., Landfester K., Mailänder V., Wurm F.R. (2016). Protein adsorption is required for stealth effect of poly(ethylene glycol)- and poly(phosphoester)-coated nanocarriers. Nat. Nanotechnol..

[53] Wilhelm C., Gazeau F., Roger J., Pons J.N., Bacri J.C. (2002). Interaction of Anionic Superparamagnetic Nanoparticles with Cells: Kinetic Analyses of Membrane Adsorption and Subsequent Internalization. Langmuir.

[54] Lesniak A., Salvati A., Santos-Martinez M.J., Radomski M.W., Dawson K.A., Aberg C. (2013). Nanoparticle adhesion to the cell membrane and its effect on nanoparticle uptake efficiency. J. Am. Chem. Soc..

[55] Tenzer S., Docter D., Rosfa S., Wlodarski A., Kuharev J., Rekik A., Knauer S.K., Bantz C., Nawroth T., Bie C. (2011). Nanoparticle size is a critical physicochemical determinant of the human blood plasma corona: A comprehensive quantitative proteomic analysis. ACS Nano.

[56] Ogawara K., Furumoto K., Nagayama S., Minato K., Higaki K., Kai T., Kimura T. (2004). Pre-coating with serum albumin reduces receptor-mediated hepatic disposition of polystyrene nanosphere: Implications for rational design of nanoparticles. J. Control. Release.

