# Supplementary material for: Limited Impact of the Protein Corona on the Cellular Uptake of PEGylated Zein Micelles by Melanoma Cancer Cells
Source: Pharmaceutics. 2022 Feb 18;14(2):439. doi: 10.3390/pharmaceutics14020439 (PMC8877401; doi:10.3390/pharmaceutics14020439)
Supplement: Supplementary file 1 [file pharmaceutics-14-00439-s001.zip › pharmaceutics-1587804-supplementary.pdf]

pharmaceutics

MDPI

Supplementary Materials: Limited Impact of the Protein Corona on the Cellular Uptake of PEGylated Zein Micelles by Melanoma Cancer Cells

Jitkasem Meewan , Sukrut Somani , Partha Laskar , Craig Irving , Margaret Mullin , Stuart Woods , Craig W. Roberts , Abdullah R. Alzahrani , Valerie A. Ferro , Suzanne McGill , Stefan Weidt , Richard Burchmore and Christine Dufès

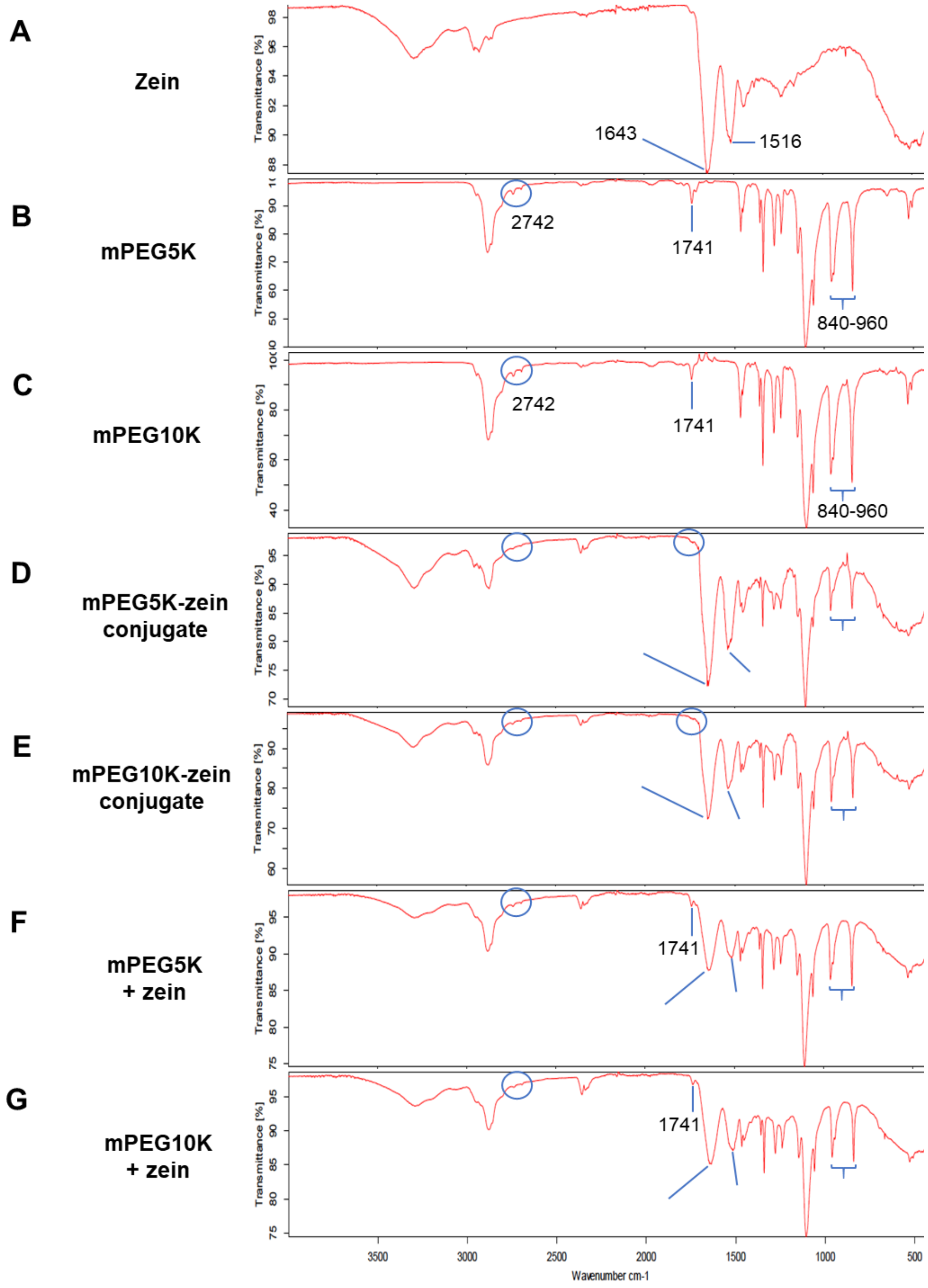

Figure S1. FTIR spectra of zein (A), mPEG5K (B), mPEG10K (C), mPEG5K-zein conjugate (D), mPEG10K-zein conjugate (E), non-conjugated mixture of mPEG5K with zein (F), and non-conjugated mixture of mPEG10K with zein (G).

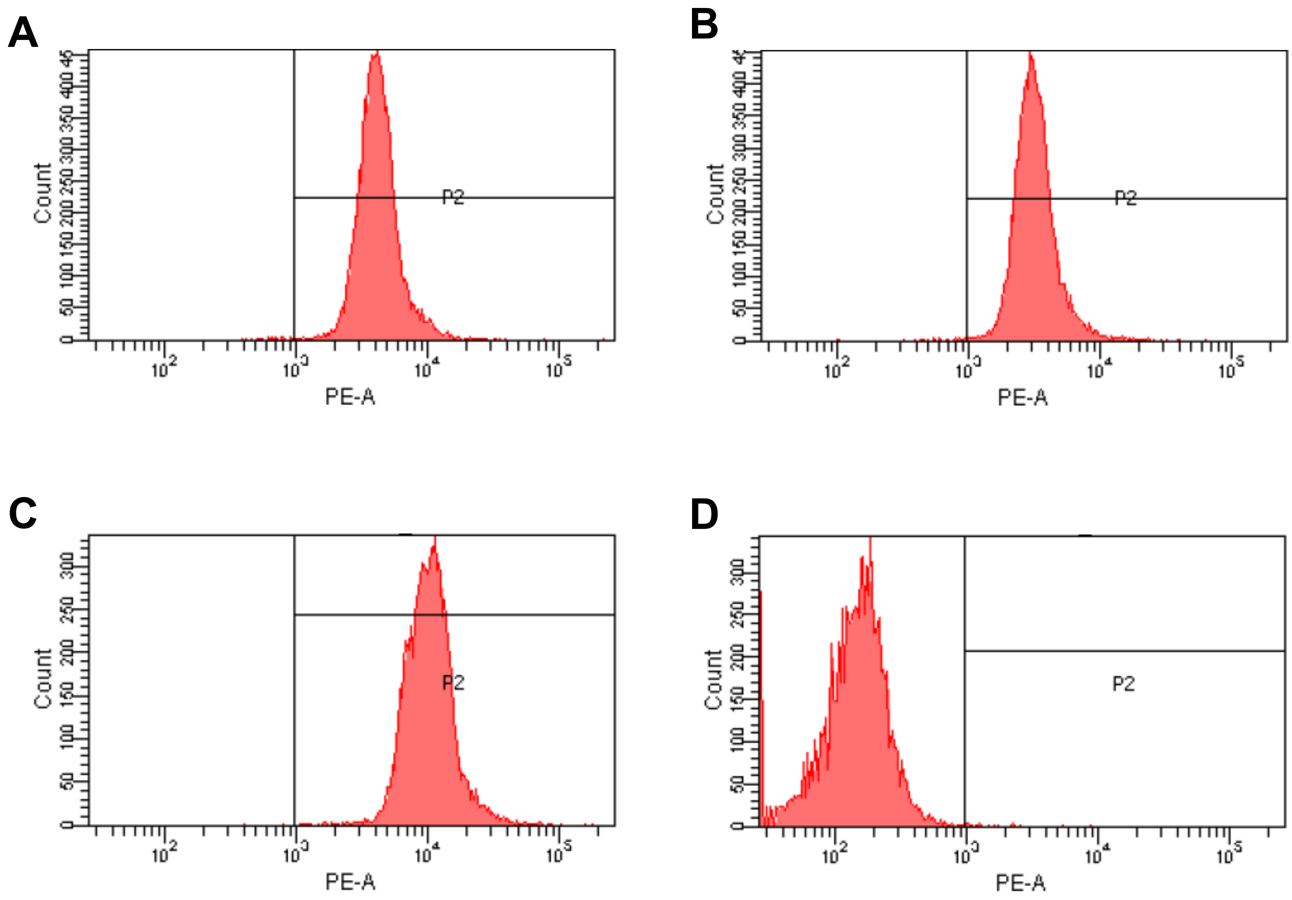

Figure S2. Flow cytometry histograms of B16-F10-luc-G5 cells following 2-h incubation with Nile red loaded in mPEG5K-zein (A) and mPEG10K-zein (B) micelles, Nile red solution (C), or left untreated (D).

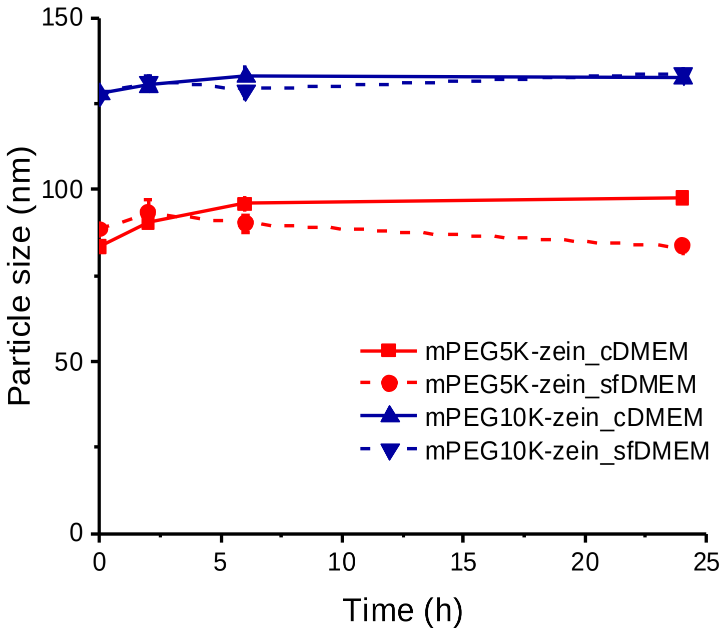

Figure S3. Size of mPEG5K-zein and mPEG10K-zein micelles in the presence or absence of 10% (v/v) FBS, over 24 h ("cDMEM": complete DMEM medium, "sfDMEM": serum-free DMEM medium) (*n* = 3).

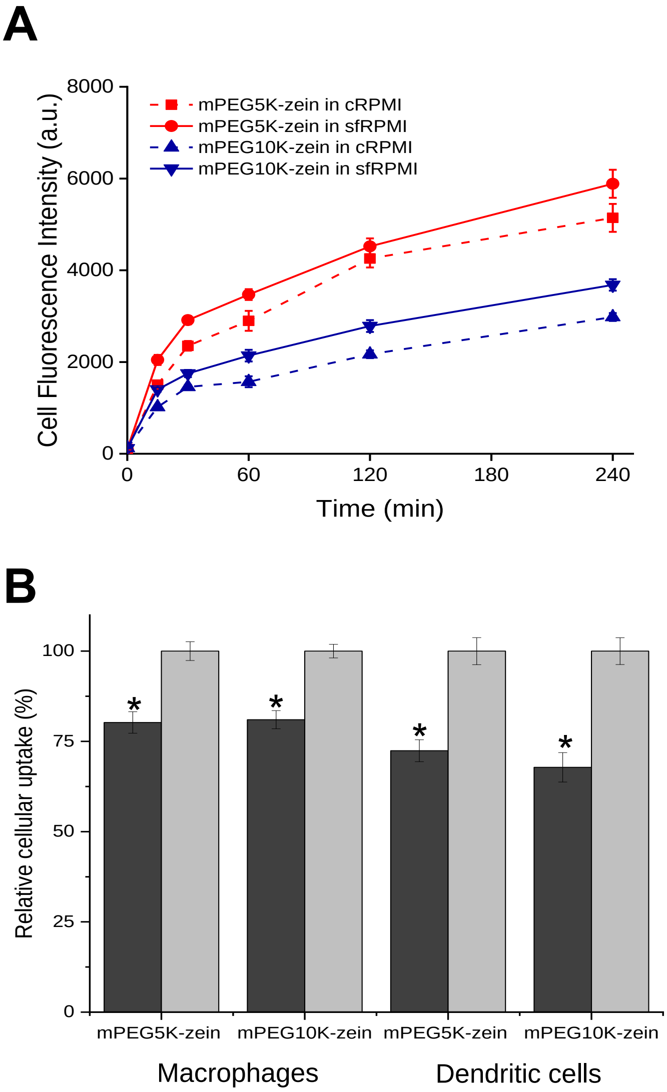

Figure S4. Effect of FBS on the cellular uptake of mPEG-zein micelles. Nile red-loaded mPEG5K-zein and mPEG10K-zein micelles were pre-incubated in complete medium or in serum-free medium at 37°C for 1 h, to allow protein adsorption to the surface of the micelles. A) Time-dependent uptake of the pre-formed corona micelles in complete and serum-free medium by B16-F10-luc-G5 cells (cRPMI: complete RPMI medium, sfRPMI: serum-free RPMI medium) (n = 3). B) Cellular uptake of the pre-formed corona micelles in cRPMI (dark gray) or sfRPMI (light gray), by macrophages and dendritic cells (n = 6) (*: $P < 0.05$, compared with sfRPMI).

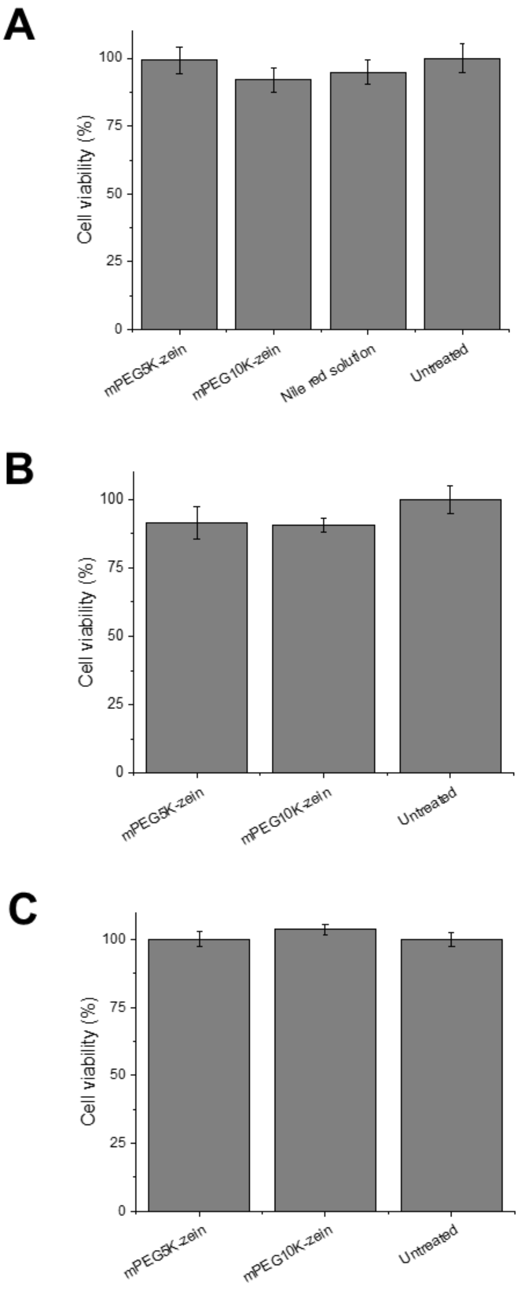

Figure S5. Viability of B16-F10-luc-G5 cancer cells (A), macrophages (B), and dendritic cells (C) treated with Nile red-loaded mPEG-zein micelles for 4 h (n = 15) (cancer cells) or 2 h (n = 10) (macrophages, dendritic cells) (controls: cells treated with Nile red solution, or left untreated). There was no statistical difference between the treatments.

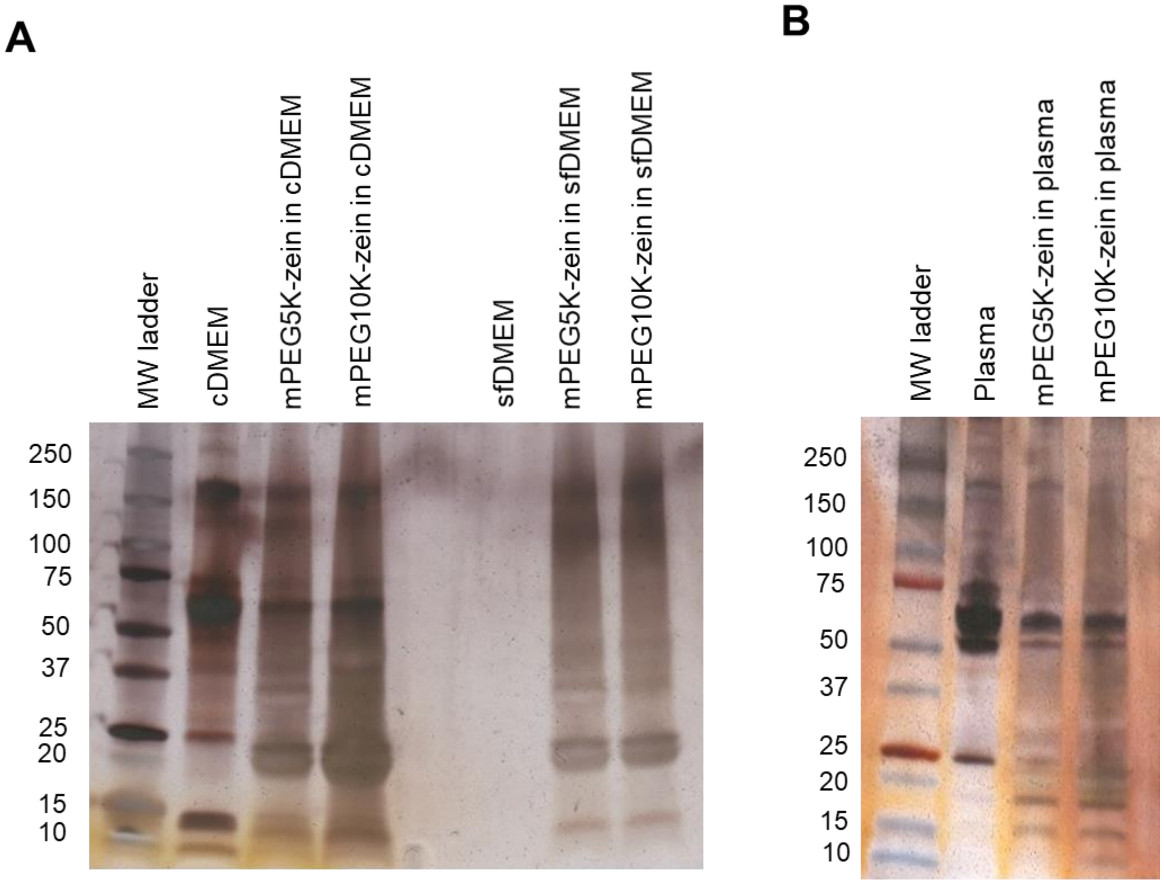

Figure S6. SDS-PAGE gels of protein corona surrounding mPEG-zein micelles following incubation in cDMEM, sfDMEM (A) and HP (B) at 37°C for 1 h. The analysis was performed in duplicate – for clarity only one replicate is shown. Protein bands at ~22-24 kDa correspond to α-zein. Other zein fractions were also detected from micelles that have been incubated with sfDMEM. However, the intensities of these bands were considerably low.

Table S1. List of hard corona proteins on mPEG5K-zein and mPEG10K-zein micelles after exposure to human plasma at 37°C for 1 h (n.d.: not detected).

Accession	Description	Mass	mPEG5K-zein		mPEG10K-zein	
			Score	emPAI	Score	emPAI
NP_000468.1	serum albumin preproprotein	71317	2765	5.74	2552	7.32
CAH18185.1	hypothetical protein (albumin)	71353	2465	4.73	2204	6.46
AAF69644.1	PRO2675 (albumin)	33466	n.d.	n.d.	2093	13.73
AFA52006.1	keratin 1	66197	2910	3.67	1097	2.93
AAG41947.1	keratin 1	66198	n.d.	n.d.	1075	2.93
NP_000414.2	keratin, type II cytoskeletal 2 epidermal	65678	1556	2.15	n.d.	n.d.
AAC83410.1	epidermal cytokeratin 2	66110	n.d.	n.d.	840	2.31
AAC41769.1	keratin type II	60448	549	0.87	n.d.	n.d.
AAA59466.1	keratin type II, partial	60258	538	0.76	n.d.	n.d.
AAH69269.1	Keratin 6A	60323	497	0.87	n.d.	n.d.
AAH24292.1	Keratin 5	62568	358	0.52	207	0.27
NP_000217.2	keratin, type I cytoskeletal 9	62255	2741	1.8	158	0.35
NP_000055.2	complement C3 preproprotein	188569	2720	0.76	1405	0.94
NP_000375.2	apolipoprotein B-100 precursor	516634	1721	0.22	812	0.27
NP_000412.3	keratin, type I cytoskeletal 10	58994	1668	1.97	1173	3.64
AAB35421.1	type I keratin 16	51548	1094	1.59	n.d.	n.d.
AAA59460.1	keratin type 16	51010	n.d.	n.d.	97	0.25
AAH02690.1	Keratin 14	51905	1009	0.92	n.d.	n.d.
NP_705694.3	keratin, type I cytoskeletal 13 isoform a	49900	300	0.35	n.d.	n.d.
BAG56970.1	unnamed protein product	62862	53	0.13	n.d.	n.d.
AMT74554.1	immunoglobulin light chain VRC01c-HuGL, partial	18550	1494	5.03	n.d.	n.d.
AMT74549.1	immunoglobulin light chain VRC01c-HuGL, partial	18437	1282	5.1	n.d.	n.d.
AMT74548.1	immunoglobulin light chain VRC01c-HuGL, partial	18337	1232	4.03	n.d.	n.d.
AAH73794.1	Unknown (protein for MGC:88814)	25236	1108	1.82	245	1.82
1RZ7_L	Chain L, Crystal Structure Of Human Anti-Hiv-1 Gp120-Reactive Antibody 48d	23285	1017	1.61	n.d.	n.d.
AAB50880.1	anitubulin IgG1 kappa VL chain {N-terminal} [human, serum, immunocytic sarcom patient PER isolate, Peptide Partial, 219 aa]	24028	1001	1.54	n.d.	n.d.
5M6A_A	Chain A, Crystal structure of cardiotoxic Bence-Jones light chain dimer H9	22803	967	1.67	n.d.	n.d.
AAF13225.1	immunoglobulin lambda light chain, partial	23197	962	2.07	n.d.	n.d.
ABU90575.2	immunoglobulin lambda 2 light chain, partial	23205	949	1.62	n.d.	n.d.
ABU90604.1	immunoglobulin kappa 1 light chain, partial	23832	944	1.55	n.d.	n.d.
AWH66747.1	immunoglobulin light chain variable region, partial	11751	758	3.71	n.d.	n.d.
BAC85363.1	unnamed protein product	54372	659	0.52	521	0.41
BAC85190.1	unnamed protein product	56528	627	0.49	n.d.	n.d.

Accession	Description	Mass	mPEG5K-zein		mPEG10K-zein	
			Score	emPAI	Score	emPAI
BAC85349.1	unnamed protein product	54127	598	0.52	n.d.	n.d.
BAC85432.1	unnamed protein product	54647	n.d.	n.d.	516	0.51
ARA90391.1	immunoglobulin heavy chain, partial	50906	524	0.81	337	0.94
BAC85202.1	unnamed protein product	53997	n.d.	n.d.	260	1.01
AAH28090.1	IGL@ protein	25119	n.d.	n.d.	248	1.83
AXN93649.1	immunoglobulin gamma 1 constant region, partial	36582	n.d.	n.d.	198	1.27
AAH70353.1	IGL@ protein	25475	n.d.	n.d.	189	1.4
AAT86037.2	immunoglobulin mu light chain variable region, partial	15959	n.d.	n.d.	168	1
ANH09850.1	immunoglobulin light chain variable region, partial	11778	n.d.	n.d.	122	0.36
P0DOX2.2	Immunoglobulin alpha-2 heavy chain BUT	49816	n.d.	n.d.	113	0.25
AAG00912.1	recombinant IgG4 heavy chain, partial	43465	n.d.	n.d.	92	0.3
AMB38464.1	immunoglobulin heavy chain variable region, partial	15667	502	1.56	n.d.	n.d.
AAD30738.1	immunoglobulin heavy chain variable region, partial	13400	n.d.	n.d.	88	1.27
AAS85877.1	immunoglobulin heavy chain, partial	16502	n.d.	n.d.	88	0.95
AIE56783.1	immunoglobulin heavy chain variable region, partial	10722	n.d.	n.d.	73	0.4
CEF92697.1	immunoglobulin heavy chain variable region, partial	11472	n.d.	n.d.	62	1.6
AAW69278.1	anti-tetanus toxoid immunoglobulin light chain variable region, partial	11764	448	1.53	n.d.	n.d.
AAF79136.1	immunoglobulin light chain variable region, partial	11739	418	0.86	n.d.	n.d.
AAB59396.1	immunoglobulin alpha-2 heavy chain, partial	37212	397	0.5	n.d.	n.d.
CAA06862.1	anti-(ED-B) scFV, partial	25357	384	0.34	n.d.	n.d.
ARA90390.1	immunoglobulin heavy chain, partial	49976	355	0.97	n.d.	n.d.
AAA19493.1	immunoglobulin kappa light chain V-Jk4, partial	12914	324	1.35	n.d.	n.d.
CAE45775.1	hypothetical protein	53011	312	0.89	n.d.	n.d.
CAC43966.1	immunoglobulin kappa light chain variable region, partial	10855	303	0.95	n.d.	n.d.
AAF79134.1	immunoglobulin light chain variable region, partial	12085	286	0.83	n.d.	n.d.
AAA69737.1	immunoglobulin light chain MRNA V-region, partial	13916	223	1.21	38	0.3
CAC85284.1	anti-peptide/MHC complex HLA-A1/MAGE-A1 monoclonal antibody heavy chain, partial	26902	221	0.74	n.d.	n.d.
AAB59394.1	immunoglobulin gamma-4 heavy chain, partial	36360	217	0.51	n.d.	n.d.
BAJ52218.1	immunoglobulin gamma heavy chain, partial	23913	213	0.87	n.d.	n.d.
CAJ75491.1	immunoglobulin lambda light chain, partial	11570	194	0.88	n.d.	n.d.
ABA00093.1	immunoglobulin epsilon heavy chain variable region, partial	13187	173	0.74	n.d.	n.d.
ABU90692.2	immunoglobulin lambda 1 light chain, partial	23106	158	0.62	n.d.	n.d.
AAY33400.1	anti-rabies virus immunoglobulin light chain variable region, partial	11887	85	0.85	n.d.	n.d.
AAD16751.1	immunoglobulin lambda light chain variable region, partial	11759	55	0.86	n.d.	n.d.

Accession	Description	Mass	mPEG5K-zein		mPEG10K-zein	
			Score	emPAI	Score	emPAI
NP_000499.1	fibrinogen alpha chain isoform alpha-E preproprotein	95656	1193	0.54	444	0.61
NP_000005.2	alpha-2-macroglobulin isoform a precursor	164614	990	0.32	763	0.38
AAA87674.1	immunoglobulin kappa light chain, partial	23727	n.d.	n.d.	683	2.5
ADX66015.1	immunoglobulin variable region, partial	15515	n.d.	n.d.	321	1.04
NP_000286.3	alpha-1-antitrypsin precursor	46878	921	0.9	926	2.33
AAA51546.1	alpha-1-antitrypsin	46787	n.d.	n.d.	925	2.33
AAH15642.1	Serpin peptidase inhibitor, clade A (alpha-1 antiproteinase, antitrypsin), member 1	46850	n.d.	n.d.	923	2.33
3V83_A	Chain A, The 2.1 Angstrom Crystal Structure Of Diferric Human Transferrin	79280	846	0.69	721	0.95
NP_000032.1	apolipoprotein E isoform b precursor	36246	n.d.	n.d.	686	1.81
EAX03569.1	hCG2001591	194140	802	0.29	1236	0.42
AAB59397.1	apolipoprotein E	36242	768	4.22	n.d.	n.d.
NP_002209.2	inter-alpha-trypsin inhibitor heavy chain H4 isoform 1 precursor	103521	527	0.29	181	0.29
NP_002017.1	fibronectin isoform 3 preproprotein	262656	501	0.06	303	0.07
NP_000574.2	vitamin D-binding protein isoform 1 precursor	54480	n.d.	n.d.	279	0.32
NP_000030.1	apolipoprotein A-I isoform 1 preproprotein	30759	480	2.81	779	4.49
AAB22835.1	apolipoprotein AI, apo AI [human, spleen, Peptide Mutant, 88 aa]	10155	199	1.93	302	5
AAA35545.1	proapo-A-I protein	30745	n.d.	n.d.	740	4.49
NP_001636.1	apolipoprotein C-I precursor	9326	n.d.	n.d.	53	0.47
P0DOX6.2	Immunoglobulin mu heavy chain OU	64244	475	0.19	n.d.	n.d.
NP_001076.2	alpha-1-antichymotrypsin precursor	47792	452	0.37	211	0.48
NP_002207.2	inter-alpha-trypsin inhibitor heavy chain H2 precursor	106853	452	0.24	139	0.15
NP_000482.3	complement C1q subcomponent subunit B precursor	26933	443	0.51	180	0.15
NP_001728.1	complement component C9 preproprotein	64615	n.d.	n.d.	63	0.06
NP_002206.2	inter-alpha-trypsin inhibitor heavy chain H1 isoform a preproprotein	101782	355	0.16	321	0.2
NP_000031.1	apolipoprotein C-III precursor	10846	330	0.4	201	1.72
AAB32200.1	apolipoprotein D, apoD	28317	314	0.93	53	0.3
NP_000168.1	gelsolin isoform a precursor	86043	259	0.14	34	0.04
AGP00859.1	immunoglobulin A heavy chain variable region, partial	14039	226	0.3	n.d.	n.d.
NP_005134.1	haptoglobin isoform 1 preproprotein	45861	215	0.64	126	0.28
AAA58902.1	Ig J-chain, partial	16041	214	0.26	75	0.26
NP_001701.2	complement factor B preproprotein	86847	209	0.09	146	0.14
EAW72575.1	alpha-1-B glycoprotein	54809	195	0.07	n.d.	n.d.
NP_000020.1	angiotensinogen preproprotein	53406	194	0.15	111	0.15
NP_000053.2	plasma protease C1 inhibitor precursor	55347	181	0.15	48	0.07

Accession	Description	Mass	mPEG5K-zein		mPEG10K-zein	
			Score	emPAI	Score	emPAI
NP_000884.1	kininogen-1 isoform 2 precursor	48936	172	0.17	55	0.17
NP_000473.2	apolipoprotein A-IV precursor	45344	172	0.39	192	0.94
AAF00489.1	hemoglobin beta subunit variant	16086	163	0.58	n.d.	n.d.
BAA00124.1	alpha-2-plasmin inhibitor precursor	54903	158	0.23	n.d.	n.d.
BAB71575.1	unnamed protein product	25941	156	0.33	172	0.54
NP_001725.1	complement C1s subcomponent isoform 1 preproprotein	78174	146	0.05	76	0.05
EAX04934.1	fibrinogen beta chain, isoform CRA_e	40167	124	0.21	n.d.	n.d.
EAX04933.1	fibrinogen beta chain, isoform CRA_d	52759	n.d.	n.d.	108	0.15
1TTC_A	Chain A, Transthyretin	13842	123	0.7	n.d.	n.d.
NP_003652.2	apolipoprotein L1 isoform a precursor	44004	111	0.09	94	0.09
NP_000479.1	antithrombin-III isoform 1 precursor	53025	110	0.15	201	0.53
CAJ75478.1	immunoglobulin heavy chain, partial	23826	105	0.17	60	0.17
NP_001822.3	clusterin preproprotein	53031	104	0.24	179	0.33
AAH34389.1	Leucine-rich alpha-2-glycoprotein 1	38372	103	0.1	65	0.22
AAI44239.1	Peptidoglycan recognition protein 2	68683	97	0.06	n.d.	n.d.
NP_000177.2	complement factor H isoform a precursor	143654	95	0.05	n.d.	n.d.
NP_006735.2	retinol-binding protein 4 isoform a precursor	23337	92	0.38	n.d.	n.d.
NP_001176.1	zinc-alpha-2-glycoprotein precursor	34465	90	0.12	63	0.12
AME15468.1	anti-HIV immunoglobulin heavy chain variable region, partial	13165	89	0.32	n.d.	n.d.
AGR34115.1	anti-HIV-1 immunoglobulin light chain variable region, partial	11208	n.d.	n.d.	44	0.38
3CU7_A	Chain A, Human Complement Component 5	189911	88	0.04	39	0.02
1I5J_A	Chain A, Nmr Structure Of Human Apolipoprotein C-Ii In The Presence Of Sds	8909	83	0.5	n.d.	n.d.
AHZ09405.1	immunoglobulin light chain variable region, partial	11334	83	0.9	n.d.	n.d.
AFQ00545.1	vitronectin, partial	25596	82	0.16	n.d.	n.d.
1JMJ_A	Chain A, Crystal Structure Of Native Heparin Cofactor Ii	55096	77	0.07	62	0.15
ANP95466.1	vitamin D binding protein, partial	3612	71	5.19	n.d.	n.d.
NP_000087.2	ceruloplasmin precursor	122997	68	0.03	28	0.03
AGP00850.1	immunoglobulin A heavy chain variable region, partial	13407	67	0.31	n.d.	n.d.
AIU95717.1	immunoglobulin kappa light chain variable region, partial	10697	66	0.41	n.d.	n.d.
AAF03677.1	apolipoprotein(a), partial	12216	66	0.35	n.d.	n.d.
AAA52173.1	serum vitamin D-binding protein precursor	54612	65	0.15	n.d.	n.d.
NP_006503.2	serum amyloid A-4 protein precursor	14851	62	0.28	n.d.	n.d.
NP_000437.3	serum paraoxonase/arylesterase 1 precursor	39877	59	0.1	93	0.1
NP_000706.1	C4b-binding protein alpha chain precursor	69042	56	0.12	n.d.	n.d.
NP_000500.2	fibrinogen gamma chain isoform gamma-A precursor	50092	47	0.08	73	0.35

Accession	Description	Mass	mPEG5K-zein		mPEG10K-zein	
			Score	emPAI	Score	emPAI
EAX01790.1	solute carrier family 9 (sodium/hydrogen exchanger), member 4	82210	44	0.05	n.d.	n.d.
NP_001637.1	apolipoprotein C-IV precursor	14886	44	0.28	n.d.	n.d.
AAS19424.1	anti-SARS S protein immunoglobulin heavy chain variable region, partial	12738	44	0.33	n.d.	n.d.
NP_000303.1	vitamin K-dependent protein C preproprotein	53406	42	0.07	n.d.	n.d.
AIZ06499.1	immunoglobulin kappa chain variable region, partial	10228	40	0.43	n.d.	n.d.
BAS02858.1	T cell receptor alpha chain V-J-region, partial	7205	37	0.64	40	0.64
NP_002640.2	phosphatidylinositol 4,5-bisphosphate 3-kinase catalytic subunit gamma isoform	127571	34	0.03	n.d.	n.d.
EAW50979.1	hCG1742973, partial	17765	31	0.23	n.d.	n.d.
EAW55766.1	hCG1984886, isoform CRA_b, partial	24205	30	0.17	n.d.	n.d.
EAX04766.1	hCG2025928	18290	29	0.22	n.d.	n.d.
AAH00539.2	JMJD1B protein, partial	173630	29	0.02	n.d.	n.d.
EAW82627.1	iduronidase, alpha-L-, isoform CRA_j	41521	27	0.09	n.d.	n.d.
CAD39181.1	hypothetical protein, partial	52942	27	0.07	n.d.	n.d.
ABO30676.1	alpha-helix coiled-coil rod homologue, partial	13127	25	0.32	n.d.	n.d.
AAB71646.1	MHC class I chain-related protein, partial	36491	23	0.11	n.d.	n.d.
NP_001013649.2	NHS-like protein 2	133831	23	0.03	n.d.	n.d.
NP_004987.2	multidrug resistance-associated protein 1	172907	22	0.02	n.d.	n.d.
AAL08624.1	NDR1-related protein NDR2	41113	21	0.1	n.d.	n.d.
CAC12843.1	lipoxygenase-3	81856	20	0.05	n.d.	n.d.
ABE97359.1	anti-Rh(D) antibody immunoglobulin heavy chain variable region, partial	14624	20	0.29	n.d.	n.d.
BAB14324.1	unnamed protein product, partial	83252	19	0.05	n.d.	n.d.
NP_001327.2	cathepsin Z preproprotein	34530	19	0.11	n.d.	n.d.
EAW64299.1	EF-hand domain family, member B, isoform CRA_a	130113	16	0.03	n.d.	n.d.
CAB44857.1	immunoglobulin mu heavy chain variable region, partial	13201	16	0.32	n.d.	n.d.
AAS01769.1	monoclonal IgM antibody heavy chain	65126	n.d.	n.d.	244	0.42
1QWH_A	Chain A, A Covalent Dimer Of Transthyretin That Affects The Amyloid Pathway	12836	n.d.	n.d.	217	2.11
NP_001677.2	ATP synthase subunit beta, mitochondrial precursor	56525	n.d.	n.d.	105	0.14
NP_000604.1	hemopexin precursor	52385	n.d.	n.d.	102	0.15
NP_001624.1	protein AMBP preproprotein	39886	n.d.	n.d.	97	0.1
NP_000629.3	vitronectin precursor	55069	n.d.	n.d.	94	0.15
AAL07469.1	alpha-1-B glycoprotein precursor	54746	n.d.	n.d.	72	0.07
AAA35952.1	beta-globin	19204	n.d.	n.d.	63	0.47
NP_001077007.1	POTE ankyrin domain family member E	122882	n.d.	n.d.	58	0.06

Accession	Description	Mass	mPEG5K-zein		mPEG10K-zein	
			Score	emPAI	Score	emPAI
NP_001630.1	serum amyloid P-component precursor	25485	n.d.	n.d.	50	0.16
NP_001613.2	alpha-2-HS-glycoprotein isoform 2 preproprotein	40114	n.d.	n.d.	50	0.1
1COH_A	Chain A, Structure Of Haemoglobin In The Deoxy Quaternary State With Ligand Bound At The Alpha Haems	15174	n.d.	n.d.	49	0.27
EAX01470.1	hCG23783, isoform CRA_a	23402	n.d.	n.d.	45	0.17
BAG06714.1	MYO5B variant protein	215218	n.d.	n.d.	45	0.02
BAG58506.1	unnamed protein product	113194	n.d.	n.d.	43	0.03
NP_689497.1	charged multivesicular body protein 4c	26394	n.d.	n.d.	38	0.15
AAG44663.1	DC33	30040	n.d.	n.d.	38	0.28
NP_003433.3	zinc finger protein 143 isoform 1 [Homo sapiens]	69709	n.d.	n.d.	36	0.06
NP_001124.1	afamin precursor	70963	n.d.	n.d.	34	0.05
SCW25082.1	Activated tyrosine kinase PDGFRB	92437	n.d.	n.d.	34	0.04
EAX06838.1	Fas (TNFRSF6) associated factor 1, isoform CRA_b	57089	n.d.	n.d.	33	0.07
XP_011524065.1	lethal(3)malignant brain tumor-like protein 4 isoform X6	60648	n.d.	n.d.	28	0.06
BAG60995.1	unnamed protein product	40215	n.d.	n.d.	27	0.1
AAM15772.1	interleukin-1 receptor associated kinase 4	51925	n.d.	n.d.	26	0.08
NP_005989.3	T-complex protein 1 subunit gamma isoform a	61066	n.d.	n.d.	22	0.06
AAG44697.1	DC37	32471	n.d.	n.d.	22	0.12
CAD38880.1	hypothetical protein	58694	n.d.	n.d.	22	0.07
EAW65837.1	tetratricopeptide repeat domain 6, isoform CRA_a	185766	n.d.	n.d.	22	0.02
XP_011536666.1	RNA-binding protein Musashi homolog 1 isoform X5	28085	n.d.	n.d.	21	0.14
AAI13880.1	SH3 domain and tetratricopeptide repeats 2	146697	n.d.	n.d.	19	0.03
BAH11896.1	unnamed protein product	17420	n.d.	n.d.	16	0.24
AAT74746.1	proteoglycan 4, partial	28812	n.d.	n.d.	15	0.14
